# Therapeutic potential of *Lycium barbarum* polysaccharide on diabetes and its associated complications: a narrative review

**DOI:** 10.3389/fnut.2025.1642252

**Published:** 2026-01-12

**Authors:** Nairong Yao, Chunguang Xie, Qiyue Yang

**Affiliations:** Department of Endocrinology, Hospital of Chengdu University of Traditional Chinese Medicine, Chengdu, Sichuan, China

**Keywords:** *Lycium barbarum* polysaccharide, diabetes, diabetic complications, molecular mechanism, therapeutic effect

## Abstract

Diabetes mellitus (DM), a complex metabolic disorder with severe complications, has been established to impose a heavy burden on patients and medical systems globally. Furthermore, it has been reported that contemporary therapeutic approaches or medications may not effectively manage DM. *Lycium barbarum* polysaccharide (LBP), a bioactive compound isolated from *Lycium barbarum* L. fruits, was shown to improve glycolipid parameters and mitigate glucotoxicity-induced target organ damage, making it a promising multifunctional hypoglycemic agent. Here, based on basic and clinical studies conducted over the past 20 years, we comprehensively review the potential benefits and molecular mechanisms of LBP in preventing and combating DM and its chronic complications. Our analysis revealed that LBP can reduce intestinal glucose digestion and absorption, improve glycolipid metabolism and insulin sensitivity, protect pancreatic *β*-cell function, inhibit oxidative stress (OS) and inflammatory responses, and regulate gut microbiota (GM), thus alleviating DM. It also exhibited significant pharmacological value in addressing the critical pathological mechanisms underlying DM-related complications. Despite the promising preclinical evidence, further exploration of LBP’s bioavailability, toxicology, structure–activity, and dose-effect relationships would still be required before clinical translation studies. We hope that our findings will lay a proper therapeutic and molecular foundation for future LBP-related research and product development in relation to treating DM and its associated complications.

## 1 Introduction

Diabetes mellitus (DM) is a complex metabolic disease characterized by a persistent chronic hyperglycemic state attributable to defective insulin secretion and impaired glucose utilization (1, 2). Owing to changes in people’s lifestyles and an aging global population, DM prevalence is presently growing at an alarming rate. According to the 11th edition of the Global Diabetes Map, ⁓589 million adults (aged 20–79 years) developed DM in 2024, a figure expected to increase to 853 million by 2050 (3). Notably, DM has been associated with multiple acute and chronic complications, including kidney failure, cardiovascular diseases (CVDs), blindness, and lower-limb amputation, among other adverse outcomes. These complications are the main manifestation of the harm caused by DM, leading to a decline in patients’ quality of life, disability, and death, while also increasing huge healthcare costs, which have imposed an enormous burden on patients and healthcare systems globally (4–8). Presently, blood glucose control is the mainstay intervention for DM and its related complications (9). Nonetheless, hypoglycemic drugs such as biguanides, sulfonylureas, and thiazolidinediones, have been associated with various side effects such as hypoglycemia, weight gain, gastrointestinal discomfort, as well as liver, kidney, and even heart injury risks. At the same time, injectable drugs are expensive and inconvenient to use (9–11). Furthermore, only a few drugs have been highlighted to exert protective effects on vital organs (12). As a result, developing improved drugs for alleviating DM is imperative.

**Figure 1:**
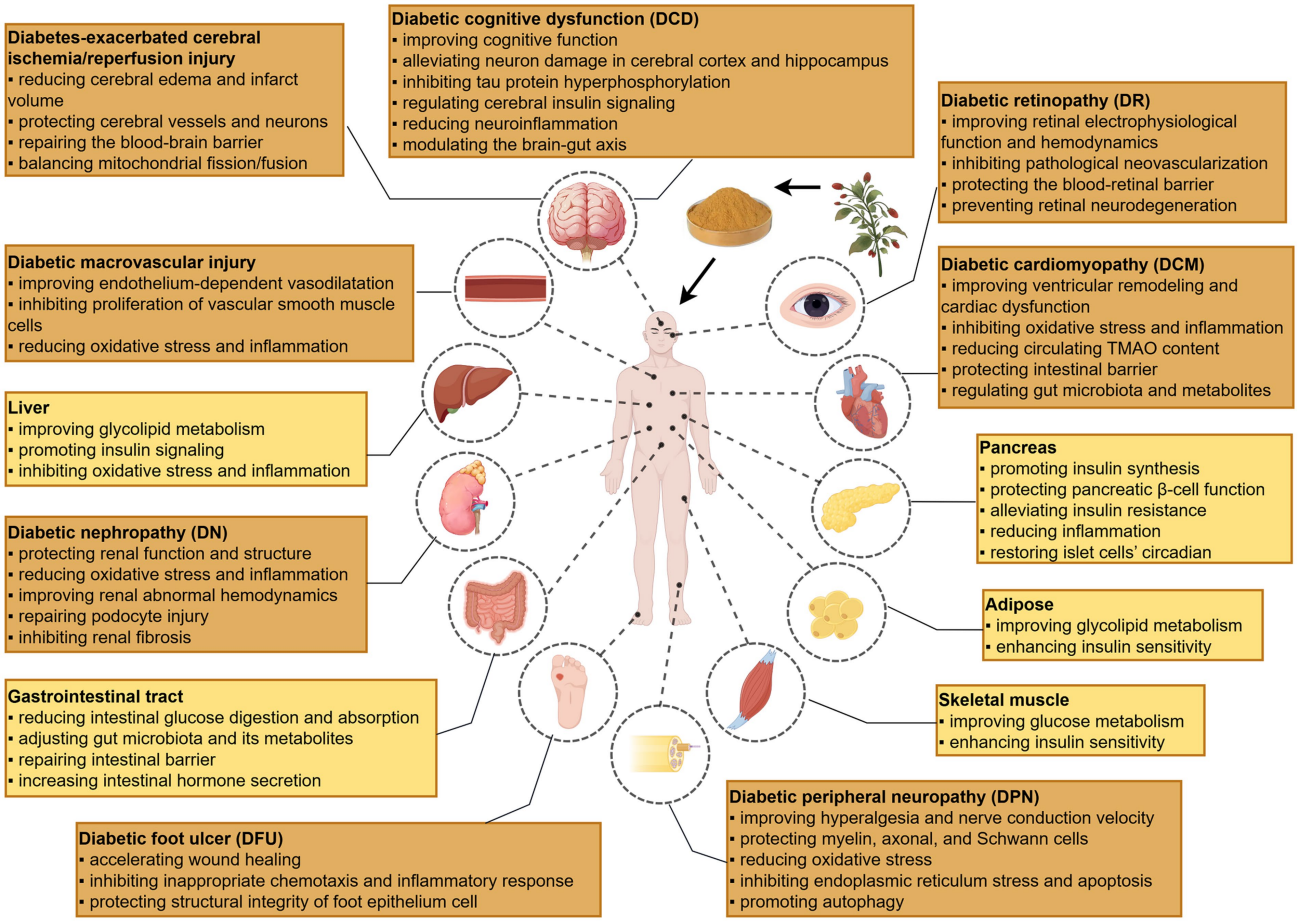
Schematic illustration of LBP’s therapeutic effects on DM and its major complications. Created with Figdraw.com.

Various functional foods and natural products have shown favorable anti-diabetic actions with wide sources and minimal side effects, highlighting their potential use as complementary and alternative therapies (13). *Lycium barbarum* L. (Solanaceae), commonly known as Goji berry or wolfberry, is a famous traditional Chinese herbal medicine that has been utilized for over 2,300 years. It is frequently employed as a popular functional food and dietary supplement to maintain and promote health (14, 15). *Lycium barbarum* polysaccharide (LBP), a water-soluble glycoconjugate extracted from *Lycium barbarum* L. fruits (LBFs), is the core bioactive component (16). According to research, LBP has demonstrated multiple pharmacological activities, including antioxidation, antitumor, immunomodulation, neuroprotection, and metabolic regulation (17, 18). Notably, increasing attention has been directed toward LBP’s therapeutic potential in DM and its associated complications. Numerous studies have reported that LBP contributes to glucose homeostasis, enhances insulin sensitivity, protects pancreatic *β*-cell function, and mitigates glucotoxicity-induced organ damage in a variety of targets and pathways (18, 19). These findings suggest that LBP may serve as a promising hypoglycemic dietary supplement or therapeutic agent. However, comprehensive evaluations regarding the therapeutic effects and molecular mechanisms underlying LBP against DM, and its nutritional translation prospects, remain limited. Therefore, this article systematically reviews preclinical and clinical investigations of LBP on DM and its seven major complications, discusses the underlying molecular mechanisms through which LBP exerts its biological activities, and outlines key directions for future research, with particular emphasis on clinical translation prospects (Figure 1). We believe this review will promote the effective utilization of LBP resources and serve as a valuable reference for advancing clinical applications and facilitating the translation of research findings into practical therapeutic strategies.

## 2 Effects of LBP on DM

There are three main categories of DM. Type 1 diabetes mellitus (T1DM), also known as insulin-dependent DM, generally results from the autoimmune destruction of insulin-producing pancreatic *β*-cells (20). On the other hand, type 2 diabetes mellitus (T2DM), formerly referred to as non-insulin-dependent DM (NIDDM), is a complex metabolic and endocrine disorder characterized by hyperglycemia, insulin resistance (IR), and relative insulin deficiency (21). The last category is gestational diabetes mellitus (GDM), a pathological state manifested as abnormal glucose tolerance or hyperglycemia attributable to insufficient insulin production or signaling transduction in pregnant women (22). Of the three categories, T2DM is the most prevalent, accounting for ⁓90% of DM cases. As a result, this review will mainly focus on the efficacy of LBP in managing T2DM.

Multiple animal and clinical studies have extensively documented LBP’s therapeutic effects on DM. For instance, in high-fat diet and streptozotocin (HFD/STZ)-induced diabetic mice, heteropolysaccharide LBP-s-1 (500 mg/kg·d, orally) for 7 weeks significantly reduced fasting blood glucose (FBG), random blood glucose (RBG), area under the curve (AUC) value of oral glucose tolerance test (OGTT), and homeostasis model assessment of insulin resistance (HOMA-IR) index; in the meantime, it also increased the serum insulin concentration (16). Additionally, in HFD/STZ-aggravated diabetic rats, crude LBP (100 mg/kg·d, orally) and its diethylaminoethyl cellulose (DEAE-cellulose) elution fraction LBP-IV (50, 100, and 200 mg/kg, orally) for 4 weeks markedly decreased FBG, postprandial blood glucose (PBG), glycated hemoglobin A1c (GHbA1c), and serum lipid levels, including triglyceride (TG), total cholesterol (TC), and low-density lipoprotein cholesterol (LDL-C) (23). Furthermore, a previous prospective, randomized, double-blind study involving T2DM patients (with a disease duration of no more than 5 years, aged 50–70 years, and baseline FBG level of approximately 7.5 mmol/L) revealed that LBP capsules (300 mg/day, orally) for 3 months substantially reduced the FBG, PBG, and serum tumor necrosis factor-*α* (TNF-α) levels, and raised the insulinogenic index (IGI) and high-density lipoprotein (HDL) level (24). Notably, patients who had not previously taken hypoglycemic drugs exhibited more obvious curative effects than those who took hypoglycemic drugs. These findings collectively highlight LBP’s potential use as a functional food ingredient for controlling glucose homeostasis. Specifically, LBP primarily exerts its anti-diabetic effects by acting on insulin-producing organs and target tissues, including the pancreas, liver, adipose tissue, and skeletal muscle. Additionally, LBP modulates the gut microbiota (GM) and regulates glucose digestion and absorption, thus influencing blood glucose levels.

### 2.1 LBP reduces intestinal glucose digestion and absorption

Suppressing glucose supply has recently gained increasing attention as the main research area in relation to DM management. According to research, inhibiting the activities of key carbohydrate hydrolases such as intestinal *α*-glucosidase and pancreatic α-amylase could delay the degradation of polysaccharides, oligosaccharides, and disaccharides in the gastrointestinal tract, thus preventing hyperglycemia (25). Kou et al. reported that LBPs-ILs extracted from ionic liquids (ILs) exerted significant inhibitory effects on both *α*-glucosidase and α-amylase in a dose-dependent manner (26). Another *in vivo* experiment involving KKAy mice revealed that LBP significantly inhibited *α*-glucosidase activity in the intestinal tract at 30 and 120 min after a meal, resulting in lower PBG levels at 30,90, and 120 min during OGTT (27). These findings suggest that LBP could be an *α*-glucosidase and α-amylase inhibitor that might antagonize glucose digestion and absorption, thus regulating the PBG peak following carbohydrate intake.

Glucose transporters, such as sodium-glucose cotransporter 1 (SGLT-1) and glucose transporter 2 (GLUT2), have been established to be responsible for the majority of intestinal glucose absorption. While SGLT1 mediates the dietary glucose uptake in the small intestine, GLUT2 mediates the release of glucose from intestinal cells into circulation (28). Notably, LBP can inhibit SGLT-1 expression in intestinal cells and decrease glucose absorption, thus reducing blood glucose concentration. LBP3b, one of the LBP sub-fractions, remarkably inhibited the glucose uptake of Caco-2 cells (human colonic cancer cell line) in a dose- and time-dependent manner to reduce PBG, a phenomenon attributable to the competition between glucose absorption and LBP3b (29). Additionally, Cai et al. revealed the underlying mechanism, reporting that LBP downregulated and competitively bound to the SGLT-1 receptor in Caco-2 cells to reduce glucose absorption in a time-dependent manner (30). Moreover, Zhao et al. found that LBP significantly decreased SGLT-1 protein expression in STC1 cells (murine entero-endocrine cell line) under high glucose conditions to alleviate DM (27).

### 2.2 LBP improves glycolipid metabolism and IR

**Figure 2:**
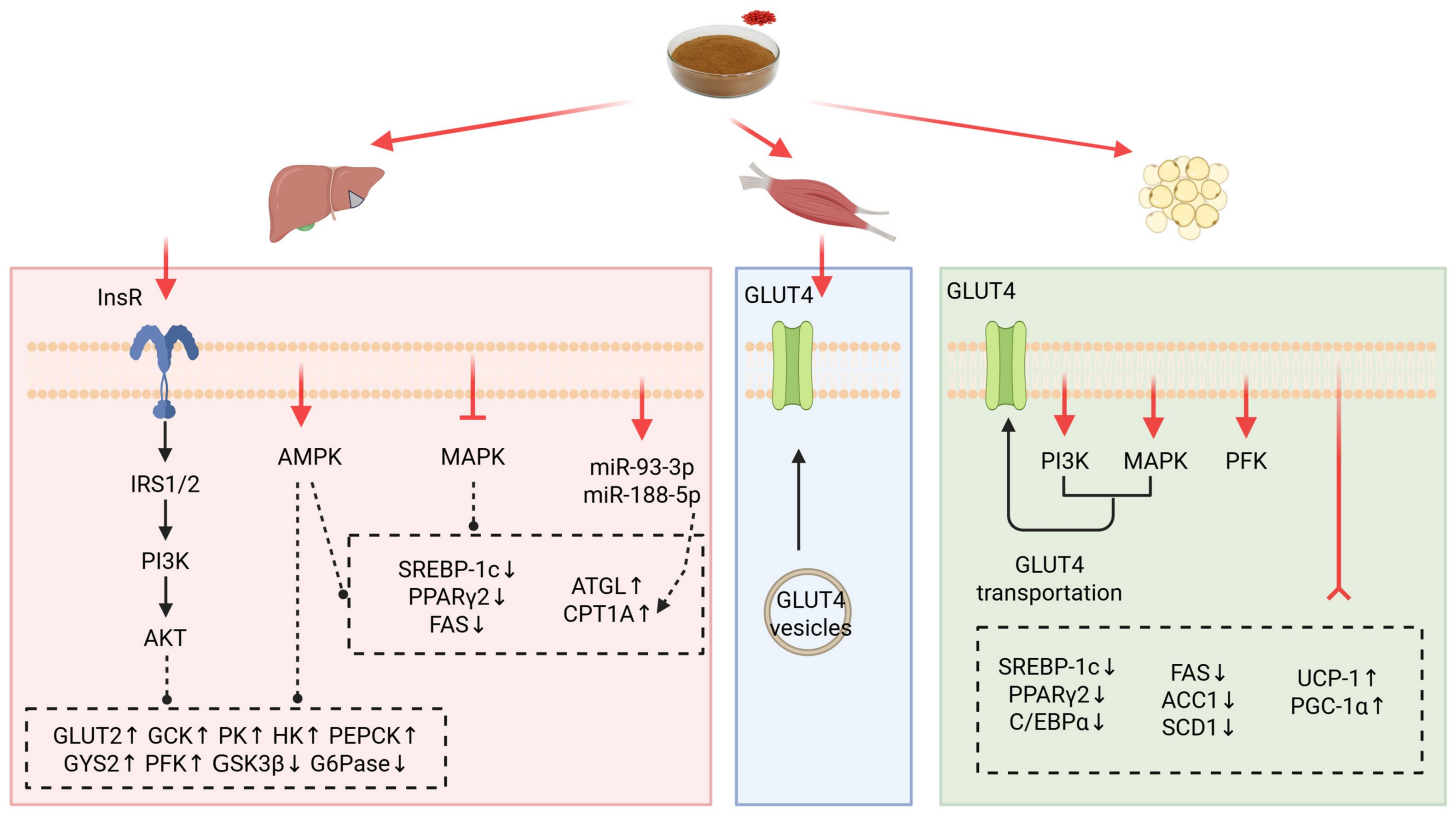
Mechanism of LBP to improve glycolipid metabolism and IR in liver, skeletal muscle, and adipose tissue. ACC1, acetyl-coenzyme A carboxylase 1; AKT, protein kinase B; AMPK, adenosine 5′-monophosphate-activated protein kinase; ATGL, adipose triacylglyceride lipase; C/EBP*α*, CCAAT/enhancer-binding protein α; CPT1A, palmitoyltransferase 1a; FAS, fatty acid synthetase; G6Pase, glucose-6-phosphatase; GCK, gluconokinase; GLUT2, glucose transporter isoform 2; GLUT4, glucose transporter isoform 4; GSK3*β*, glycogen synthase kinase 3β; GYS2, glycogen synthase 2; HK, hexokinase kinase; InsR, insulin receptor; IRS-1, insulin receptor substrate-1; IRS-2, insulin receptor substrate-2; MAPK, mitogen-activated protein kinase; PEPCK, phosphoenolpyruvate carboxykinase; PFK, phosphofructokinase; PGC-1α, peroxisome proliferator-activated receptor-*γ* coactivator-1α; PI3K, phosphatidylinositol 3-kinase; PK, pyruvate kinase; PPARγ2, peroxisome proliferator-activated receptor γ2; SCD1, stearoyl-coenzyme A desaturase 1; SREBP-1c, sterol regulatory element-binding protein-1c; UCP-1, uncoupling protein-1. Created with BioRender.com.

Peripheral tissues, particularly insulin-sensitive tissues such as the liver, adipose tissue, and skeletal muscle might exhibit an inadequate response to the physiological effects of circulating insulin, a phenomenon known as IR (31). Evidence suggests that insufficient insulin reactivity and sensitivity could lead to an imbalance between glucose utilization and hepatic glucose output, impairing glucose homeostasis, thus resulting in T2DM occurrence (32). In this regard, it is noteworthy that LBP is often employed as an insulin sensitizer in treating IR and DM (Figure 2; Table 1).

**Table 1:** Effects and mechanisms of LBP against DM by improving IR, oxidative stress, and inflammatory response.

Animal/Cell	Method of modeling	Type of model	Dosage and duration	Described effects	Potential mechanism	References
C57BL/6 J mice	HFD	IR	100 mg/kg·d, 24 weeks, orally	Serum glucose↓, insulin↓, IPGTT↓, IPITT↓	Liver GCK↑, PK↑, PEPCK↓, G6Pase↓, GSK3β↑, IRS-1↑, PI3K↑, AKT↑; liver MCP-1↓, IL-6↓, TNF-α↓, ROS↓, SOD↑, CAT↑, GSH↑, GSSG↓, GSH/GSSG↑, p-Nrf2↑, HO-1↑, p-JNK↓	(34)
HepG2 cells	palmitate	IR	100–600 μg/mL, 12 h	Glycogen concentration↑, glucose production↓	GSK3β↑, IRS-1↑, PI3K↑, AKT↑; SOD↑, CAT↑, p-Nrf2 (nuclear translocation)↑, HO-1↑, p-JNK↓	
SD rats	HFD	IR	1 mg/kg·d, 4–12 weeks, orally	BW↓, GTT↓, ITT↓	Liver resistin↓, GSK3α↓, IRS-1↑, SREBP-1c ↓, PPARγ2↓, ATGL↑, adiponectin ↑, serum FFA; liver CAT↑, GSH-Px↑, MDA↓, NTR↓, TNF-α↓, IL-1β↓, COX-2↓, MCP-1↓, caspase-3↓, Bax↓, Bcl-2↑; p-p38 MAPK↓, p-JNK↓, p-ERK1/2↓, NF-κB p65↓, IκBα↑, mTOR↓, p62↑, beclin 1↑, Atg5↑, LC3-II↑	(35)
C57BL/6 J mice	HFD + STZ	T2DM	25, 100, 500 mg/kg·d, 7 weeks, orally	FBG↓, OGTT↓, FINS↓, HOMA-IR↓	Liver HK↑, PK↑	(16)
HepG2 cells	High glucose	T2DM	15.63, 31.25, 62.5, 125, 250 nmol/L, 24 h	Glucose consumption↑	/	
3 T3-L1 cells	High glucose	T2DM				
C57BL/6 J mice	HFD + STZ	T2DM	80 mg/kg·d, 8 weeks, orally	/	Liver GLUT2↑; hepatic glycogen↑, GYS2↑; PEPCK↓, G6Pase↓; GK↑, PK↑, PFK↑; GCK↑, p-AMPK↑	(37)
HepG2 cells	High glucose, insulin	T2DM	10, 25, 50, 100 μg/mL, 24 h	Glucose consumption and uptake↑	p-GSK3↑, p-AKT↑; ROS↓	
SD rats	HFD + STZ	T2DM	50, 100, 200 mg/kg·d, 30 days, orally	BW↑, UV↓, FBG↓, GHbA1c↓, FINS↑, HOMA-IR↓	Hepatic glycogen↑, the citrate cycle, alanine, aspartate and glutamate metabolism, glyoxylate and dicarboxylate metabolism↑	(38)
3 T3-L1 cells	Insulin, dexamethasone, isobutylmethylxanthine	IR	300 μg/mL, 24 h	Glucose uptake↑, lipid accumulation↓	PFK↑; ACC1↓, FAS↓	(39)
OLETF rats	Standard rat chow	NIDDM	10 mg/kg·d, 4 weeks, orally	BW↓, serum glucose↓, insulin↓	Adipocytes 2-deoxyglucose uptake↑, GLUT4 activity and translocation↑, PI3K↑, p38 MAPK↑	(40)
Wistar rats	HFD + STZ	T2DM	10 mg/kg·d, 3 weeks, orally	BW↓, OGTT↓, FINS↓, ISI↑, TC↓, TG↓	Skeletal muscle GLUT4 activity and translocation↑	(41)
C57BL/6 J mice	HFD	IR	100 mg/kg·d, 24 weeks, orally	BW↓, liver weight↓, fat weight↓, serum glucose↓, TC↓, TG↓, LDL-C↓, HDL-C↑, NEFA↑	Liver FAS↓, SREBP-1c↓, CPT1A↑, p-AMPK↑; adipose tissue UCP-1↑, PGC-1α↑	(43)
ICR mice	HFD	IR	0.2%, 10 weeks, orally	BW↓, liver weight↓, fat weight↓, serum TC↓, TG↓, LDL-C↓, HDL-C↑, liver TC↓, TG↓	Serum and liver MDA↓; adipose tissue ACC1↓, FAS↓, SCD1↓, SREBP-1c↓, PPARγ↓, C/EBPα↓	(44)
3 T3-L1 cells	Insulin, dexamethasone, isobutylmethylxanthine	IR	25,50,100, 200 μg/L, 24 h	Lipid accumulation↓	PPARγ↓, C/EBPα↓, FAS↓	(45)
3 T3-L1 cells	Insulin, dexamethasone, isobutylmethylxanthine	IR	50,100, 200, 400 μg/mL, 24 h	Lipid accumulation↓	PPARγ↓, C/EBPα↓, FAS↓, SREBP-1↓, leptin↓, adiponectin ↑	(46)
C57BL/6 J mice	HFD	GDM	150 mg/kg·d, 6 weeks, orally	OGTT↓, serum TC↓, TG↓, LDL-C↓, HDL-C↑	CPT1A ↑, miR-93-3p↑, miR-188-5p↑	(48)
C57BL/6 J mice	HFD + STZ	T2DM	50,100,200 mg/kg·d, 6 weeks, orally	FBG↓, OGTT↓, ITT ↓, GHbA1c↓; INS↑, HOMA-IR↓, HOMA-β↑; TC↓, TG↓, LDL-C↓, HDL-C↑	Serum TAOC↑, liver TAOC↑, CAT↑, SOD↑, GSH↑, GSH-Px↑, MDA↓; serum and liver IL-6↓, IL-1β↓, TNF-α↓, LPS↓; liver InsR↑, IRS-1↑, IRS-2↑, PI3K↑, AKT↑, GLUT2↑, PEPCK↓, liver glycogen↑, muscle glycogen↑; morphology and quantity of pancreatic β-cells↑; GM changes, intestinal mucosa↑	(62)
Wistar rats	STZ	DM	50,100,200 mg/kg·d, 30 days, orally	BW↑, BG↓, INS↑, TC↓, TG↓, LDL-C↓, HDL-C↑	Serum SOD↑, MDA↓; liver SOD↑, CAT↑, GSH-Px↑, GR↑, MDA↓	(63)
SD rats	HFD + STZ	T2DM	400 mg/kg·d, 8 weeks, orally	BW↓, FBG↓, GHbA1c↓, TC↓, TG↓	Serum IL-10↑, IL-1β↓, IL-6↓, IL-17A↓, TNF-α↓; GM changes, intestinal mucosa↑	(64)
C57BL/6 J mice	HFD + STZ	T2DM	100,200 mg/kg·d, 6 weeks, orally	FBG↓, GHbA1c↓, INS↑	Serum LPS↓, IL-6↓, TNF-α↓, IFN-γ↓, liver TLR4↓, TNF-α↓, IL-6↓, IFN-γ↓, pancreas IL-6↓, IFN-γ↓; morphology and quantity of pancreatic β-cells↑; GM changes, intestinal mucosa↑	(65)

ACC1: acetyl-coenzyme A carboxylase 1; AKT: protein kinase B; Atg5: autophagy-related 5 homolog; ATGL: adipose triacylglyceride lipase; Bax: Bcl-2-associated X protein; Bcl-2: B-cell lymphoma-2; BW: body weight; C/EBPα: CCAAT/enhancer-binding protein α; caspase-3: cysteine aspartate-specific protease-3; CAT: catalase; COX-2: cyclooxygenase-2; CPT1A: palmitoyltransferase 1a; FAS: fatty acid synthetase; FBG: fasting blood glucose; FFA: free fatty acids; FINS: fasting insulin; G6Pase: glucose-6-phosphatase; GCK: gluconokinase; GDM: gestational diabetes mellitus; GHbA1c: glycated hemoglobin A1c; GK: glucokinase; GLUT2: glucose transporter isoform 2; GLUT4: glucose transporter isoform 4; GM: gut microbiota; GR: glutathione reductase; GSH: glutathione; GSH-Px: glutathione peroxidase; GSK3α: glycogen synthase kinase 3α; GSK3β: glycogen synthase kinase 3β; GSSG: oxidized glutathione; GTT: glucose tolerance test; GYS2: glycogen synthase 2; HDL-C: high-density lipoprotein cholesterol; HFD: high-fat diet; HK: hexokinase; HO-1: heme oxygenase 1; HOMA-IR: homeostasis model assessment of insulin resistance; HOMA-β: homeostasis model assessment of β-cell function; IFN-γ: interferon-γ; IL-1β: interleukin-1β; IL-6: interleukin-6; INS: insulin; InsR: insulin receptor; IPGTT: intraperitoneal glucose tolerance test; IPITT: intraperitoneal insulin tolerance test; IR: insulin resistance; IRS-1: insulin receptor substrate-1; IRS-2: insulin receptor substrate-2; ISI: insulin sensitive index; ITT: insulin tolerance test; IκBα: inhibitor of nuclear factor-κB α; LC3-II: microtubule-associated protein light chain 3-II; LDL-C: low-density lipoprotein cholesterol; LPS: lipopolysaccharide; MCP-1: monocyte chemoattractant protein-1; MDA: malondialdehyde; mTOR: mammalian target of rapamycin; NEFA: non-esterified fatty acids; NF-κB: nuclear factor-κB; NIDDM: non-insulin-dependent diabetes mellitus; NTR: nitrotyrosine; OGTT: oral glucose tolerance test; OLETF: Otsuka Long-Evans Tokushima Fatty; p38 MAPK: p38 mitogen-activated protein kinase; p62: sequestosome 1; p-AMPK: phosphorylated adenosine 5’-monophosphate-activated protein kinase; PEPCK: phosphoenolpyruvate carboxykinase; p-ERK1/2: phosphorylated extracellularly regulated kinase1/2; PFK: phosphofructokinase; PGC-1α: peroxisome proliferator-activated receptor-γ coactivator-1α; PI3K: phosphatidylinositol 3-kinase; p-JNK: phosphorylated c-Jun N-terminal kinase; PK: pyruvate kinase; p-Nrf2: phosphorylated nuclear factor-E2-related factor 2; p-p38 MAPK: phosphorylated p38 mitogen-activated protein kinase; PPARγ2: peroxisome proliferator-activated receptor γ2; ROS: reactive oxygen species; SCD1: stearoyl-coenzyme A desaturase 1; SOD: superoxide dismutase; SREBP-1c: sterol regulatory element-binding protein-1c; STZ: streptozotocin; T2DM: type 2 diabetes mellitus; TAOC: total antioxidant capacity; TC: total cholesterol; TG: triglyceride; TNF-α: tumor necrosis factor-α; UCP-1: uncoupling protein-1; UV: volume of urine in 24 h.

The liver, the key insulin target organ, is crucially involved in glycemic excursion regulation in the body (33). In HFD-induced IR mice livers and palmitate (PA) -induced HepG2 cells (human hepatocellular carcinoma cell line), LBP enhanced the phosphorylation of insulin receptor substrate-1 (IRS-1), phosphatidylinositol 3-kinase (PI3K), and protein kinase B (AKT), thus promoting insulin signaling transduction (34). Furthermore, hepatic glucose consumption, hepatic glycogen content and downstream glycogen synthase kinase 3β (GSK3β) phosphorylated protein expression increased, implying that LBP promoted liver glucose uptake and glycogen synthesis. Additionally, LBP reduced the mRNA transcription of the downstream gluconeogenic key enzyme genes phosphoenolpyruvate carboxykinase (PEPCK) and glucose-6-phosphatase (G6Pase), ameliorating the glycemic disorder. Xiao et al. also demonstrated that LBP regulated the IRS-1/GSK3α pathway in the liver of IR rats, thus improving IR and glucose metabolism (35). Insulin-triggered glycolysis is considered the simplest and most fundamental aspect of nutrient metabolism (36). Notably, LBP upregulated the activity of hepatic glycolytic enzymes, including hexokinase (HK), gluconokinase (GCK), and pyruvate kinase (PK), thereby improving blood glucose levels in the HFD/STZ-induced diabetic model or HFD-induced IR model (16, 34). More recently, LBP has been found to activate the phosphorylation of adenosine 5′-monophosphate-activated protein kinase (AMPK), a key metabolic regulator, thereby comprehensively modulating glucose uptake, glycogen synthesis, and gluconeogenesis/glycolysis flux to improve IR (37). Importantly, findings from a previous untargeted metabolomics study demonstrated that LBP also regulates tricarboxylic acid (TCA) cycle, alanine, aspartate, and glutamate metabolism, and glyoxylate and dicarboxylate metabolism in diabetic rats’ livers, thus exerting anti-diabetic effects (38).

Besides the liver, adipose tissue and skeletal muscle are the other key insulin target tissues. In these tissues, the impairment of glucose uptake and utilization has been closely associated with IR (33). For instance, Zhu and Chung et al. reported that glucose consumption and the glycolysis key enzyme phosphofructokinase (PFK) of 3 T3-L1 preadipocytes were markedly upregulated after treatment with different concentrations of LBP (16, 39). Furthermore, Zhao et al. reported that the purified fraction LBP-4a promoted the translocation and activation of glucose transporter isoform 4 (GLUT4) and 2-deoxyglucose (2-DG) uptake in epididymal adipose tissue, thereby alleviating IR in Otsuka Long-Evans Tokushima Fatty (OLETF) rats. This phenomenon could be attributed to the regulation of PI3K and p38 mitogen-activated protein kinase (p38 MAPK) activities (40). Moreover, Zhao et al. found that LBP facilitated GLUT4 translocation in NIDDM rats, stimulating glucose uptake in skeletal muscle and decreasing fasting insulin (FINS) and PBG levels in OGTT, ultimately dramatically raising the insulin sensitive index (ISI) (41). These findings collectively suggest that LBP could modulate intracellular insulin signaling and multiple links of glucose metabolism in insulin-sensitive tissues, thereby relieving IR and hyperglycemia.

Impaired glucose metabolism can disrupt lipid homeostasis, leading to increased lipolysis, triglyceride accumulation, LDL-C levels, and decreased HDL-C, which might contribute to the release of free fatty acids (FFA), adipocytokines, and inflammatory cytokines such as TNF-*α* and interleukin-6 (IL-6). Increased systemic FFA and cytokine fluxes subsequently affect peripheral tissues’ insulin sensitivity, block the insulin signaling pathway, and disturb glucose consumption, further exacerbating IR and T2DM (42). Beyond its direct effects on glucose metabolism, LBP also demonstrates a profound ability to modulate lipid metabolism. LBP can eliminate ectopic lipid accumulation and accelerate fatty acid oxidative decomposition, potentially alleviating IR and abnormal glucose tolerance. Extensive researches have since demonstrated that LBP regulated the activity of key transcription factors and rate-limiting enzymes associated with lipid metabolism in the liver and adipose tissues: (1) the key molecule in biological energy metabolism, AMPK; (2) the lipogenesis-relevant nuclear receptors, sterol regulatory element-binding protein-1c (SREBP-1c) and peroxisome proliferator-activated receptors (PPARs), and the key regulator of adipocyte differentiation, CCAAT/enhancer-binding protein *α* (C/EBPα); (3) the lipogenesis key enzymes, acetyl-coenzyme A carboxylase (ACC), fatty acid synthetase (FAS), and stearoyl-coenzyme A desaturase (SCD); (4) the key enzyme for adipose lipid mobilization, adipose triacylglyceride lipase (ATGL), and the enzyme of fatty acids *β*-oxidation, palmitoyltransferase 1a (CPT1A); (5) the nuclear receptor coactivator of energy expenditure, peroxisome proliferator-activated receptor-*γ* coactivator-1α (PGC-1α), and its downstream uncoupling protein-1 (UCP-1) (35, 39, 43–45). Changes in these crucial factors resulted in LBP’s four notable therapeutic effects: (1) inhibited denovo lipogenesis, lipid droplet accumulation, and mature adipocyte formation; (2) increased oxidative decomposition of fatty acids; (3) stimulated adipose tissue browning and thermogenic expenditure; (4) reduced serum FFA content, which effectively alleviated lipid toxicity-aggravated IR. Moreover, LBP intervention could modulate several adipocytokines including adiponectin, resistin, and leptin, impacting insulin sensitivity and energy balance (35, 46).

In addition to the regulation of key metabolic enzymes and transcription factors, emerging evidence suggests that LBP’s beneficial effects on glycolipid metabolism are also mediated through epigenetic mechanisms, particularly involving microRNAs (miRNAs), which have been widely documented to exert a specific effect in maintaining glucose homeostasis (47). For instance, Xiao et al. reported that LBP could markedly upregulate miR-93-3p and miR-188-5p, promoting CPT1A protein expression, thus enhancing *β*-oxidation of hepatic fatty acids, ultimately reversing IR and abnormal glucose tolerance in HFD-induced GDM mice (48).

### 2.3 LBP restores pancreatic *β*-cell function

**Figure 3:**
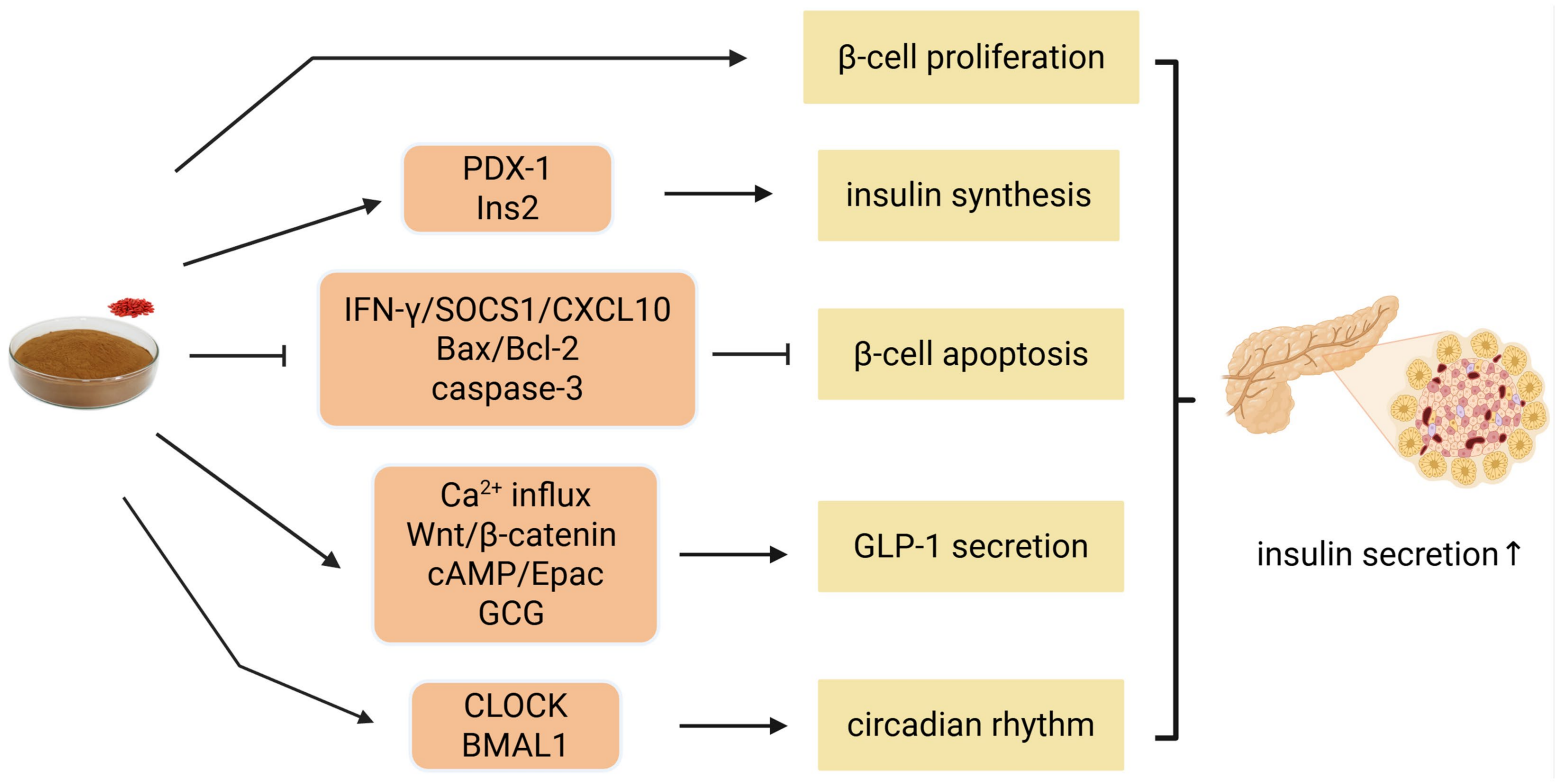
Mechanism of LBP to restore pancreatic β-cell function. Bax, Bcl-2-associated X protein; Bcl-2, B-cell lymphoma-2; BMAL1, brain and muscle Arnt-like protein-1; cAMP/Epac, cyclic adenosine monophosphate/exchange protein directly activated by cAMP; caspase-3, cysteine aspartate-specific protease-3; CLOCK, circadian locomotor output cycles kaput; CXCL10, C-X-C motif chemokine ligand 10; GCG, glucagon gene; GLP-1, glucagon-like peptide-1; IFN-γ, interferon-γ; Ins2, insulin 2; PDX-1, pancreas duodenal homeobox 1; SOCS1, suppressor of cytokine signaling 1. Created with BioRender.com.

Insulin, the sole hypoglycemic hormone in the body secreted by pancreatic β-cells, is crucially involved in glucose homeostasis (49). Insulin deficiency resulting from mass loss and failure of pancreatic β-cell function is the core pathophysiological mechanism underlying DM onset (50). Moreover, LBP was shown to improve the quality and quantity of pancreatic β-cells, promoting insulin secretion via multiple protective mechanisms, thus alleviating DM (Figure 3).

LBP supports normoglycemia in part by stimulating *β*-cell proliferation and insulin synthesis. Zou and Zhu et al. confirmed that in RIN-m5F cells (rat insulinoma cell line), LBP counteracted alloxan-induced damage and facilitated cell neogenesis in a dose- and time-dependent manner, leading to enhanced insulin secretion (16, 51). Similarly, Liu et al. found that in HFD/STZ-induced T2DM rats, LBP up-regulated pancreas duodenal homeobox 1 (PDX-1) mRNA, a key transcription factor involved in *β*-cell differentiation and insulin gene activation, which augmented insulin production and stabilized glucose levels (52, 53). Li et al. further reported that LBP increased insulin 2 (Ins2) gene expression and improved mature β-cell function, helping maintain adequate β-cell mass (54).

In addition to promoting β-cell growth, LBP inhibits apoptosis to preserve islet function. In SJ β-cells (immortalized pancreatic *β*-cell line), LBP attenuated interferon-*γ* (IFN-γ)-induced apoptosis by downregulating the downstream suppressor of cytokine signaling 1 (SOCS1) and C-X-C motif chemokine ligand 10 (CXCL10) (54). LBP also modulated apoptosis-related proteins in H₂O₂-stimulated INS-1 cells, elevating B-cell lymphoma-2 (Bcl-2) while reducing Bcl-2-associated X protein (Bax) and cysteine aspartate-specific protease-3 (caspase-3) levels, thereby decreasing the apoptotic rate and restoring insulin secretion (55).

Beyond these direct effects, LBP influences glucagon-like peptide-1 (GLP-1) secretion and circadian rhythm regulation—two key mechanisms associated with pancreatic *β*-cell protection. GLP-1, a peptide hormone secreted by intestinal L-cells, promotes the transdifferentiation of islet *α*-cells into β-cells, stimulates β-cell proliferation, inhibits apoptosis, and enhances insulin synthesis, thereby exerting comprehensive protective effects on β-cell function (56). Zhao et al. demonstrated that LBP enhances both first-phase GLP-1 release via stimulating Ca^2+^ influx and second-phase secretion via regulating the Wnt/β-catenin and cyclic adenosine monophosphate/exchange protein directly activated by cAMP (cAMP/Epac) pathways, along with upregulating the glucagon gene (GCG) expression (27). Moreover, circadian rhythm disruption has been increasingly recognized as a contributing factor to the development of DM (57). Mutations in clock genes such as the circadian locomotor output cycles kaput (CLOCK) and brain and muscle Arnt-like protein-1 (BMAL1) can accelerate *β*-cell failure (58). Notably, LBP-4a was shown to modulate CLOCK, BMAL1, and melatonin receptor type 2 (MT2) expression in diabetic rats, leading to normalized insulin secretion rhythms and improved β-cell function (59).

Collectively, these findings illustrate that LBP preserves β-cell function through a coordinated mechanism involving direct cytoprotection, enhanced GLP-1 signaling, and restoration of circadian regulation.

### 2.4 LBP reduces oxidative stress and inflammation

Oxidative stress (OS) and inflammation are closely linked to IR and pancreatic β-cell dysfunction, playing a critical role in the pathogenesis of DM. These events also constitute key factors in the progression of DM. It seems that hyperglycemia increases the production of reactive oxygen species (ROS) through multiple mechanisms, creating an OS environment that directly causes damage to tissues and cellular structures and functions. Furthermore, ROS activates downstream signaling pathways, including c-Jun N-terminal kinase (JNK), p38 MAPK, extracellularly regulated kinase1/2 (ERK1/2), and nuclear factor-κB (NF-κB), leading to the release of proinflammatory cytokines such as IL-1β, IL-6, IL-8, IL-12, IL-18, TNF-*α*, and IFN-*γ*, which interfere with insulin signaling and promote β-cell apoptosis. In turn, these inflammatory mediators, particularly ILs and TNF-α, amplify OS, thereby perpetuating a self-sustaining “ROS-inflammation-DM” vicious cycle (60, 61). LBP exerts anti-diabetic effects, potentially attributable to their antioxidant and anti-inflammatory properties, as well as their regulatory effects on various cellular signaling pathways.

Evidence indicated that LBP could enhance the body’s antioxidant defense capacity by regulating the nuclear factor-E2-related factor 2/antioxidant response element/heme oxygenase 1 (Nrf2/ARE/HO-1) pathway and autophagy, thereby improving OS in IR and diabetic models (34, 35). Specifically, LBP significantly increased the serum total antioxidant capacity (T-AOC) and superoxide dismutase (SOD) content, elevated hepatic SOD, catalase (CAT), glutathione (GSH), glutathione peroxidase (GSH-Px), and glutathione reductase (GR) levels, which are major components of the defense system against ROS (34, 35, 62, 63). At the same time, LBP markedly reduced serum and hepatic levels of malondialdehyde (MDA), a lipid peroxidation product, and scavenged hepatic ROS and nitrotyrosine (NTR), established biomarkers of oxidative damage (34, 35, 62, 63). Improvements in these indicators can effectively protect insulin target organs and the pancreas from oxidative injury, ensuring the orderly progression of glucose and lipid metabolism and insulin secretion, thus contributing to the mitigation of DM. Additionally, studies have confirmed that LBP can inhibit the phosphorylation of p38 MAPK, JNK, and ERK1/2, and suppress the NF-κB-mediated transcription, thereby decreasing the production of proinflammatory factors including IL-1β, IL-6, TNF-α, monocyte chemoattractant protein-1 (MCP-1), and cyclooxygenase-2 (COX-2) in an IR model (34, 35). This phenomenon promoted the recovery of hepatic insulin signaling and glucose utilization. Moreover, LBP upregulated anti-inflammatory factor IL-10 and downregulated proinflammatory factors IL-1β, IL-6, IL-17A, TNF-α, and IFN-γ, ameliorating systemic inflammation and reducing pancreatic cell apoptosis in a DM model (62, 64, 65). This anti-inflammatory effect could be attributed to the modulation of GM-mucosal immunity-inflammation-DM axis (62, 64, 65). Overall, LBP may serve as an effective antioxidant and anti-inflammatory agent in the management of DM.

### 2.5 LBP regulates GM and repairs the intestinal barrier

As earlier mentioned, a GM disorder might impact intestinal barrier function, inducing metabolic endotoxemia and chronic low-grade inflammation, ultimately causing IR, obesity, and DM (66). Multiple studies have highlighted GM regulation and intestinal barrier protection as the key mechanisms in LBP-based DM treatment. For instance, Lu et al. reported that LBP improved GM diversity, reversed DM-induced changes in both *Firmicutes* and *Bacteroidetes*, promoted the growth of beneficial bacteria (e.g., *Bifidobacterium*, *Lactobacillus*, and *Alistipes*), and reduced the relative abundance of pathogenic bacteria (e.g., *Blautia* and *Desulfovibrio*) in diabetic rats. Furthermore, LBP activated intestinal mucosa Toll-like receptor 2 (TLR2) intraepithelial γδ T cells and upregulated intestinal tight junctions (TJs) such as zonula occludens-1 (ZO-1) and occludin, potentially repairing the intestinal barrier and inhibiting enteral and parenteral inflammation, ultimately improving DM-related biochemical abnormalities (64). Moreover, Liu et al. demonstrated that LBO, a degraded version of LBP, increased the abundance of beneficial bacteria (e.g., *Lactobacillus*, *Bacteroides*, *Prevotella*, and *Akkermansia*), alleviated intestinal mucosal edema and inflammatory infiltration, and reduced the serum diamine oxidase (DAO) and D-lactic acid (D-LA) content, improving the compactness of the intestine in HFD/STZ-induced diabetic mice, ultimately relieving DM symptoms (67). Additionally, Zhou et al. found that in a prediabetic state, the purified fraction LBPs-4 markedly increased the intestinal goblet cell count, ameliorated intestinal epithelial injury, and elevated mucin 2 (MUC2) expression, thus promoting the structural recovery of the intestinal mucus layer, and subsequently reducing the production and translocation of lipopolysaccharide (LPS) and maintaining glucose homeostasis (68).

**Figure 4:**
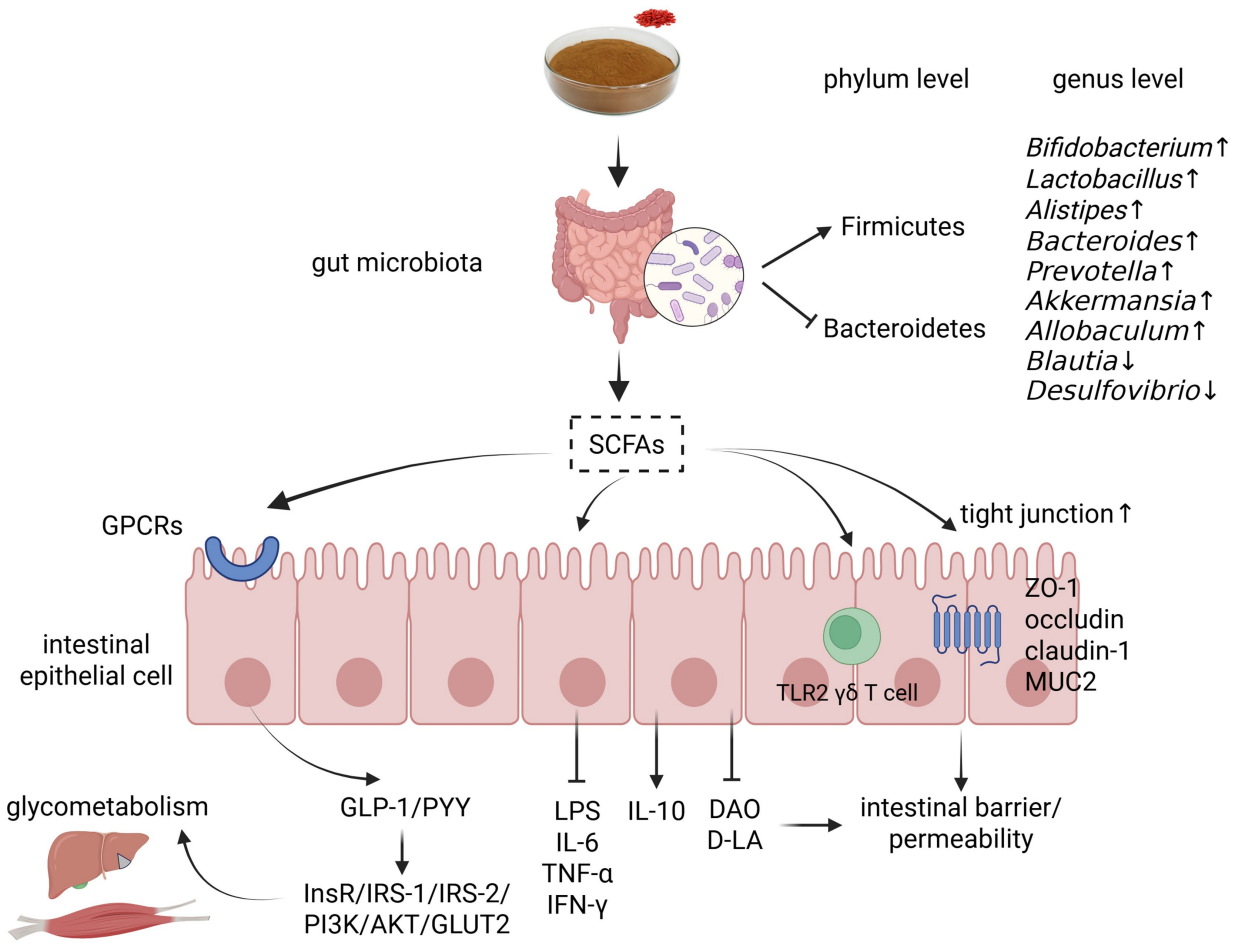
Regulatory mechanism of GM by LBP in DM treatment. AKT, protein kinase B; DAO, diamine oxidase; D-LA, D-lactic acid; GLP-1, glucagon-like peptide-1; GLUT2, glucose transporter isoform 2; GPCRs, G protein-coupled receptors; IL-10, interleukin-10; IL-6, interleukin-6; InsR, insulin receptor; IFN-γ, interferon-γ; IRS-1, insulin receptor substrate-1; IRS-2, insulin receptor substrate-2; LPS, lipopolysaccharide; MUC2, mucin 2; PI3K, phosphatidylinositol 3-kinase; SCFAs, short chain fatty acids; TLR2 γδ T cell, Toll-like receptor 2 intraepithelial γδ T cell; TNF-α, tumor necrosis factor-α; ZO-1, zonula occludens-1. Created with BioRender.com.

As the main fermentation products of GM, short chain fatty acids (SCFAs), including acetate, propionate, butyrate, and valerate, can provide energy for the intestinal epithelium, strengthen the intestinal barrier function, regulate systemic inflammation, and remodel host metabolism (69). Notably, these SCFAs are an important link between GM and DM, and reversing DM-induced SCFA downregulation could improve metabolic disturbance and promote the recovery of glycolipid parameters. In diabetic mice, LBP significantly increased fecal SCFA production in a dose-dependent manner, as well as upregulated the relative expression of the colon G protein-coupled receptor (GPCR) 41, GPCR43, peptide YY (PYY), and GLP-1, thus activating the hepatic insulin receptor (InsR)/IRS-1/IRS-2/PI3K/AKT/GLUT2 signaling pathway, decreasing PEPCK transcription, and augmenting the liver and skeletal muscle glycogen concentration (62). Furthermore, Zhou et al. found that LBP increased the relative abundance of butyrate producer *Allobaculum* and increased total SCFA and N-butyrate concentration in fecal and cecal contents, subsequently promoting colonic TJ and MUC2 protein expression and decreasing the systemic inflammatory response, thus alleviating pancreatic islet atrophy and *β*-cell apoptosis and reducing blood glucose levels in diabetic mice (65). Moreover, LBP could upregulate acetic acid secretion, potentially attenuating duodenal hyperconstriction, and ultimately improving glucose and lipid metabolism in prediabetic mice (70). Overall, LBP can regulate the bacterial and metabolite components of GM, maintain intestinal mucosal barrier integrity and permeability, and promote the “microbiota-SCFAs-GPCRs/GLP-1/PYY-glycemic metabolism” cascade reaction, thus preventing and curing DM (Figure 4).

## 3 Effects of LBP on diabetic complications

Persistent hyperglycemia could result in various microvascular and macrovascular complications, including the impairment of the function of the heart, brain, kidneys, and other important organs, severely affecting diabetic patients’ living quality and prognosis. In this context, it is noteworthy that LBP could ameliorate these complications through multiple pathways (Table 2).

**Table 2:** Effects and mechanisms of LBP against diabetic complications.

Disease	Animal/Cell model	Dosage and duration	Described effects	Potential mechanism	References
Diabetic nephropathy	Wistar rats; STZ	50,100,200 mg/kg·d, 30 days, orally	BW↑, BG↓, INS↑, TC↓, TG↓, LDL-C↓, HDL-C↑	Serum SOD↑, MDA↓; kidney SOD↑, CAT↑, GSH-Px↑, GR↑, MDA↓	(62)
Diabetic nephropathy	Wistar rats; STZ	10 mg/kg·d, 4 weeks, orally	BG↓, TC↓, TG↓; BUN↓, urine proteins↓, Scr↓, KW/BW↓, Ccr↓	Kidney SOD↑, CAT↑, GSH-Px↑, GST↑, GSH↑, MDA↓, PKC↓, ERK1/2↓	(72)
Diabetic nephropathy	SD rats; HFD + STZ	100,250,500 mg/kg·d, 4 weeks, orally	FBG↓, OGTT↓, INS↑; BUN↓, urine albuminuria↓	Serum SOD↑, GSH-Px↑, IL-2↓, IL-6↓, TNF-α↓, IFN-α↓, MCP-1↓, ICAM-1↓, kidney NF-κB↓	(73)
Diabetic nephropathy	SD rats; STZ	1.6 g/kg·d, 8 weeks, orally	FBG↓; BUN↓, Scr↓, urine microalbumin↓, renal interstitial fibrosis↓	Serum HA↓, LN↓, PC-III↓, C-IV↓, kidney Notch1↓, Jagged1↓, Hes1↓	(74)
Diabetic nephropathy	Japanese white rabbits; HFD + alloxan	10 mg/kg·d, 4–12 weeks, orally	KW/BW↓, podocyte structure↑	Kidney Nephrin↑	(75)
Diabetic nephropathy	C57BL/6 J mice; HFD + STZ	40,80,160 mg/kg·d, 8 weeks, orally	FBG↓, HOMA-IR↓; BUN↓, Scr↓, urine microalbumin↓	Kidney TNF-α↓, IL-1β↓, IL-6↓, SAA3↓, IκBα↑, nuclear translocation of NF-κB p65↑	(77)
Diabetic nephropathy	Glomerular mesangial cells; high glucose	200, 400, 800 mg/L, 48 h	Mesangial cell proliferation↓	ROS↓, PKC↓, TGF-β1↓, FN↓	(78)
Diabetic nephropathy	SD rats; HFD + STZ	20,40,80 mg/kg·d, 4 weeks, orally	GHbA1c↓; glomerular morphology↑	Serum AGEs↓, kidney IL-8↓	(79)
Diabetic nephropathy	Japanese white rabbits; HFD + alloxan	10 mg/kg·d, 4–12 weeks, orally	FBG↓; BUN↓, Scr↓, urine proteins↓, KWI↓	Kidney Ang II↓, MCP-1↓, ICAM-1↓, NF-κB↓	(82)
Diabetic retinopathy	SD rats; STZ	200, 400 mg/kg·d, 20 weeks, orally	ERG (a-wave, b-wave, OPs) ↑, retinal blood flow (PSV, EDV, CRV, MV) ↑, retina morphology↑	Retinal GFAP↓, VEGF↓, PEDF↑	(89)
Diabetic retinopathy	RF/6A cells; high glucose	600 mg/L, 48 h	RF/6A cell proliferation↓, angiogenesis↓	miR-15a-5p↑, ASM↑, VEGFA↓, VEGFR2↓, ANG1↑, ANG2↓	(90)
Diabetic retinopathy	ARPE-19 cells; high glucose, H_2_O_2_	100, 200,400 μg/L, 24 h	ARPE-19 cell proliferation↑	SOD↑, CAT↑, MMP2↓, Bax↓	(92)
Diabetic retinopathy	SD rats; STZ	250 mg/kg·d, 12 weeks, orally	BG↓, TC↓, TG↓; EB infiltration↓; retina morphology↑	Retinal ROCK1↓, p-MLC↓, p-Occludin↑	(93)
	RF/6A cells; high glucose	1 g/L, 1, 3, 5,7 day	RF/6A cell permeability and apoptosis↓	ROCK1↓, p-MLC↓, p-Occludin↑	
Diabetic retinopathy	SD rats; STZ	6%, 24 weeks, orally	RGC morphology↑, mitochondrial pathological changes↓, apoptosis↓	Retinal SOD↑, MDA↓, VEGF↓	(96)
Diabetic peripheral neuropathy	SD rats; HFD + STZ	250, 1,000 mg/kg·d, 10 weeks, orally	FBG↓; MNCV↑	Sciatic nerve MDA↓, GSH-Px↑	(99)
Diabetic peripheral neuropathy	SD rats; HFD + STZ	250, 1,000 mg/kg·d, 10 weeks, orally	FBG↓; sciatic nerve morphology↑	Sciatic nerve GRP78↓, PERK↓, CHOP↓, Bax↓, Bcl-2↑, Bcl-2/Bax↑	(100)
Diabetic peripheral neuropathy	SD rats; STZ	500 mg/kg·d, 12 weeks, orally	FBG↓; mechanical allodynia↓, thermal hyperalgesia↓, SNCV↑, SNAP↑	Sciatic nerve P0↑, MBP↑; mTOR↓, p-mTOR↓, p70S6K↓, p-p70S6K↓, LC3-II↑, Beclin 1↑, P62↓	(101)
Diabetic peripheral neuropathy	RSC96 cells; high glucose	100, 200, 400, 800 μg /mL, 48 h	Schwan cell viability↑	/	(102)
Diabetic macroangiopathy	SD rats; STZ	30, 60 mg/kg·d, 12 weeks, orally	FBG↓; EDV↑	Thoracic aorta Calpain-1↓, eNOS↑, NO↑	(110)
Diabetic macroangiopathy	A7r5 cells; high glucose	60 80,100 μg/mL, 72 h	A7r5 cell proliferation↑	/	(111)
Diabetic macroangiopathy	SD rats; HFD + STZ	20, 40 mg/kg·d, 4 weeks, orally	GHbA1c↓, INS↑, TG↓, LDL-C↓; vascular endothelial structure↑	Serum IL-6↓, IL-4↑, SOD↑, GSH-Px↑; ICAM-1↓, p38 MAPK↑	(112)
Diabetic macroangiopathy	SD rats; STZ + tMCAO	50 mg/kg·d, 4 weeks, orally	FBG↓; cerebral edema↓, infarct volume↓, IgG extravasation↓, neurological deficits scores↓	CoW ICA’ CSA↑, eNOS↑, α-SMA↑; morphology and density of cerebral parenchymal capillaries↑; neuronal protection↑	(115)
Diabetic macroangiopathy	SD rats; STZ + tMCAO	25 mg/kg·d, 4 weeks, orally	FBG↓; survival rate↑, infarct volume↓, neurological deficits scores↓, IgG extravasation, neuronal apoptosis↓	BBB structure↑, ZO-1↑, occludin↑, claudin-5↑	(114)
Diabetic macroangiopathy	SD rats; STZ + tMCAO	25 mg/kg·d, 4 weeks, orally	FBG↓; infarct volume↓, neurological deficits scores↓, pyknotic cells↓	Cerebral Opa1↑, p-Drp1/Drp1↓, p-Drp1↓, Drp1↓	(116)
Diabetic cardiomyopathy	SD rats; STZ	30, 60 mg/kg·d, 12 weeks, orally	FBG↓; LVSP↓, LVEDP↓, HW/BW↓, serum ANP↓, BNP↓	Serum TNF-α↓, IL-6↓; cardiac superoxide anion↓, eNOS↑, iNOS↓, TNF-α↓, IL-6↓, ICAM-1↓, VCAM-1↓, TLR4↓, NF-κB↓, Calpain-1↓	(120)
Diabetic cardiomyopathy	C57BL/6 J mice; HFD	100 mg/kg·d, 2 months, orally	Serum GLC↓, INS↓, TC↓, TG↓, LDL-C↓, HDL-C↓; left ventricular systolic and diastolic function↓	Serum TMAO↓, TNF-α↓, IL-17A↓, MDA↓; GM changes, intestinal mucosa↑	(124)
Diabetic cognitive dysfunction	Wistar rats; HFD + STZ	50, 100 mg/kg·d, 10 weeks, orally	Escape latency↓, target quadrant residence time↑	Brain p-Tau↓, PI3K/AKT/GSK3β↓	(127)
	Primary cortical neurons extracted from SD pregnant mice; High glucose	40 μM, 24 h	The axon transport of injured neurons↑	p-Tau↓, PI3K/AKT/GSK3β↓	
Diabetic cognitive dysfunction	C57BL/6 J APP/PS1 transgenic mice; HFD + STZ	100 mg/kg·d, 3 months, orally	Escape latency↓, platform crossing times↑, target quadrant residence time↑, swimming distance↑	Brain p-Tau↓, p-GSK3β↓	(128)
Diabetic cognitive dysfunction	C57BL/6 J mice; HFFD	200 mg/kg·d, 14 weeks, orally	BW↓, FBG↓, OGTT↓, GHbA1c↓, FINS↓, HOMA-IR↓, LDL-C↓; escape latency↓, platform crossing times↑, target quadrant residence time↑	Serum LPS↓, TNF-α↓, IL-6↓; brain Iba-1↓, GFAP↓, NLRP3↓, TNF-α↓, BDNF↑, PSD-95↑; GM changes, intestinal mucosa↑, SCFAs↑, GPCRs↑	(131)
Diabetic foot ulcer	Rats; HFD + STZ, thin iron pressing	14 days, orally	Wound healing rate↑	Serum IL-6↑, wound CXCL12↓, CXCR4↓; wound Nephrin↑	(137)

Abbreviation: AGEs: advanced glycosylation end products; AKT: protein kinase B; Ang II: angiotensin II; ANG1: angiogenin 1; ANG2: angiogenin 2; ANP: atrial natriuretic peptide; ASM: acid sphingomyelinase; Bax: Bcl-2-associated X protein; BBB: blood-brain barrier; Bcl-2: B-cell lymphoma-2; BDNF: brain-derived neurotrophic factor; BG: blood glucose; BNP: brain natriuretic peptide; BUN: blood urea nitrogen; BW: body weight; C-IV: type IV collagen; CAT: catalase; Ccr: creatinine clearance rate; CHOP: CCAAT/enhancer-binding protein homologous protein; CoW: circle of Willis; CRV: central retinal vein velocity; CSA: cross-sectional area; CXCL12: chemokine (CXC motif) ligand 12; CXCR4: chemokine (CXC motif) receptor 4; EB: Evans Blue; EDV: mean end-diastolic velocity; eNOS: endogenous nitric oxide synthase; ERG: electroretinogram; ERK1/2: extracellularly regulated kinase1/2; FBG: fasting blood glucose; FINS: fasting insulin; FN: fibronectin; GFAP: glial fibrillary acidic protein; GHbA1c: glycated hemoglobin A1c; GLC: glucose; GM: gut microbiota; GPCRs: G protein-coupled receptors; GR: glutathione reductase; GRP78: glucose-regulated protein 78; GSH: glutathione; GSH-Px: glutathione peroxidase; GSK3β: glycogen synthase kinase 3β; GST: glutathione transferase; HA: hyaluronic acid; HDL-C: high-density lipoprotein cholesterol; HFD: high-fat diet; HFFD: high-fat and high-fructose diet; HOMA-IR: homeostasis model assessment of insulin resistance; HW/BW: heart weight/body weight; Iba-1: ionized calcium-binding adapter molecule-1; ICA: internal carotid artery; ICAM-1: intercellular adhesion molecule-1; IFN-α: interferon-α; IL-17A: interleukin-14A; IL-1β: interleukin-1β; IL-2: interleukin-2; IL-4: interleukin-4; IL-6: interleukin-6; IL-8: interleukin-8; iNOS: inducible nitric oxide synthase; INS: insulin; IκBα: inhibitor of nuclear factor-κB α; KW/BW: kidney weight/body weight; KWI: kidney weight index; LC3-II: microtubule-associated protein light chain 3-II; LDL-C: low-density lipoprotein cholesterol; LN: laminin; LPS: lipopolysaccharide; LVEDP: left ventricular end-diastolic pressure; LVSP: left ventricular systolic pressure; MBP: myelin basic protein; MCP-1: monocyte chemoattractant protein-1; MDA: malondialdehyde; MMP2: matrix metalloproteinase 2; MNCV: motor nerve conduction velocity; mTOR: mammalian target of rapamycin; MV: mean velocity; NF-κB: nuclear factor-κB; NLRP3: NOD-like receptor thermal protein domain-associated protein 3; NO: nitric oxide; OGTT: oral glucose tolerance test; Ops: oscillatory potentials; P0: myelin protein zero; p38 MAPK: p38 mitogen-activated protein kinase; P62: sequestosome 1; p70S6K: p70 ribosomal protein S6 kinase; PC-III: type III procollagen; p-Drp1: phosphorylated Drp1; PEDF: pigment epithelium derived factor; PERK: protein kinase R-like endoplasmic reticulum kinase; PI3K: phosphatidylinositol 3-kinase; PKC: protein kinase C; p-MLC: phosphorylated myosin light chain; p-mTOR: phosphorylated mammalian target of rapamycin; p-Occludin: phosphorylated Occludin; p-p70S6K: phosphorylated p70 ribosomal protein S6 kinase; PSD-95: postsynaptic density protein-95; PSV: mean systolic peak velocity; p-Tau: phosphorylated Tau; RGC: retinal ganglion cell; ROCK1: RhoA-associated protein kinase 1; ROS: reactive oxygen species; SAA3: serum amyloid A3; SCFAs: short chain fatty acids; Scr: serum creatinine; SNAP: sensory nerve action potential; SNCV: sensory nerve conduction velocity; SOD: superoxide dismutase; STZ: streptozotocin; TC: total cholesterol; TG: triglyceride; TGF-β1: transforming growth factor-β1; TLR4: Toll-like receptor 4; TMAO: trimethylamine-N-oxide; tMCAO: transient middle cerebral artery occlusion; TNF-α: tumor necrosis factor-α; VCAM-1: vascular adhesion molecule-1; VEGF: vascular endothelial growth factor; VEGFA: vascular endothelial growth factor A; VEGFR2: vascular endothelial growth factor receptor 2; ZO-1: zonula occludens-1; α-SMA: α-smooth muscle actin.

### 3.1 LBP and diabetic nephropathy

**Figure 5:**
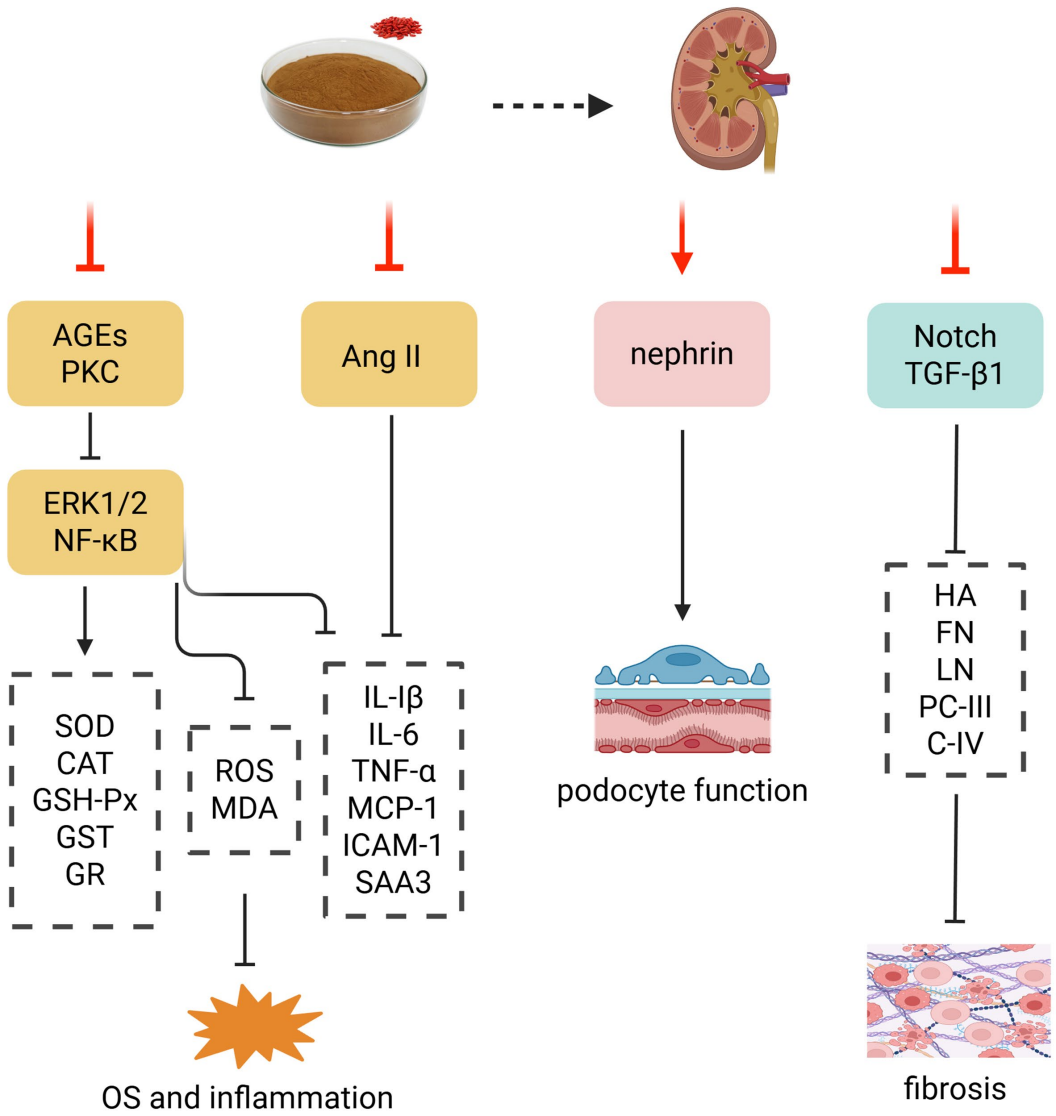
Effect and mechanism of LBP in treating DN. AGEs, advanced glycosylation end products; Ang II, angiotensin II; CAT, catalase; C-IV, type IV collagen; ERK1/2, extracellularly regulated kinase1/2; FN, fibronectin; GR, glutathione peroxidase; GSH-Px, glutathione peroxidase; GST, glutathione transferase; HA, hyaluronic acid; ICAM-1, intercellular adhesion molecule-1; IL-1β, interleukin-1β; IL-6, interleukin-6; LN, laminin; MCP-1, monocyte chemoattractant protein-1; MDA, malondialdehyde; NF-κB, nuclear factor-κB; OS, oxidative stress; PC-III, type III procollagen; PKC, protein kinase C; ROS, reactive oxygen species; SAA3, serum amyloid A3; SOD, superoxide dismutase; TGF-β1, transforming growth factor-β1; TNF-α, tumor necrosis factor-α. Created with BioRender.com.

Characterized by microalbuminuria and a slow and sustained decline in renal function that eventually progresses to end-stage renal disease (ESRD), diabetic nephropathy (DN) is the most common microvascular complication of DM (71). LBP has been demonstrated to mitigate glucotoxicity, OS, inflammation, and renal fibrosis, while repairing podocyte injury. These effects collectively contribute to a reduction in key renal function indicators, such as serum creatinine (SCr), blood urea nitrogen (BUN), and 24 h urinary protein, by 20–41.1% compared to baseline levels, as well as amelioration of renal histopathological alterations, thus effectively preserving renal function and retarding DN progression (72–75) (Figure 5).

Hyperglycemia-induced OS and inflammatory responses were found to be key factors in DN onset and progression (76). Notably, LBP could counteract these processes by suppressing ROS and MDA levels, enhancing antioxidant enzymes [e.g., SOD, CAT, GSH-Px, glutathione transferase (GST), and GR], and decreasing proinflammatory cytokines [e.g., IL-1β, IL-2, IL-6, IL-8, TNF-*α*, interferon α (IFN-α), MCP-1, intercellular adhesion molecule-1 (ICAM-1) and serum amyloid A3 (SAA3)] in the DN (63, 72, 73, 77–79). Mechanistically, LBP inhibited the advanced glycosylation end products (AGEs) formation and polyol and protein kinase C (PKC) pathway activation to reduce the generation of ROS; as well as inhibited the ERK1/2 phosphorylation, inhibitor of nuclear factor-κB α (IκBα) degradation, and nuclear translocation of NF-κB p65 to interrupt the downstream inflammatory cascade, ultimately improving DM-induced renal inflammation (72, 73, 75, 77, 78).

In the early stages, typical pathological changes of DN include glomerular hyperfiltration and hyperperfusion, which have been closely associated with renin-angiotensin system (RAS) activation and angiotensin II (Ang II) overexpression in diabetic patients, leading to DN onset (80). Furthermore, Ang II might stimulate mononuclear macrophages to release chemokines, cytokines, and adhesion molecules, inducing chronic inflammation and promoting DN onset (81). LBP has been demonstrated to reduce Ang II, MCP-1, and ICAM-1 concentration in the renal tissue of DN rabbits, as well as the kidney weight index (KWI), implying that LBP can improve the abnormal hemodynamics of the nephron and alleviate renal inflammatory hypertrophy (82).

Nephrin, a glomeruli podocyte slit diaphragm protein, plays a critical role in maintaining podocyte morphology and function (83), and its deficiency could lead to structural and functional damage in podocytes and the slit diaphragm, primarily causing massive albuminuria formation and disease progression in DN patients (83). In previous research, LBP was shown to significantly upregulate the transcription level and protein expression of nephrin, reduce podocyte swelling and foot process fusion, ameliorate pathological damage to the podocyte ultrastructure, and preserve the filtration function of glomerular basement membrane (GBM) in DN rabbits (75).

Renal fibrosis, the principal pathological manifestation of end-stage DN, could result in loss of renal function in DN patients (84). Multiple studies have shown that the Notch and transforming growth factor-β1 (TGF-β1)/Smad3 pathways could trigger epithelial-mesenchymal transition (EMT) and extracellular matrix (ECM) accumulation, leading to renal interstitial fibrosis (85). LBP demonstrated its value in reducing the proportion of renal collagen fibers via inhibiting Notch1/Jagged1/Hes1 pathway and decreasing serum levels of hyaluronic acid (HA), laminin (LN), type III procollagen (PC-III), and type IV collagen (C-IV) in STZ-induced DN rats (74). Furthermore, under high glucose conditions, LBP downregulated the expression of TGF-β1 and fibronectin (FN) in glomerular mesangial cells, preventing the progression of renal interstitial fibrosis (78). As a result, LBP holds promise for future clinical application in the treatment of DN and may impede its progression through multiple mechanisms.

### 3.2 LBP and diabetic retinopathy

Diabetic retinopathy (DR), an ocular microvascular complication of DM, is the most common cause of vision loss among the working-age population globally (86). Its main pathological features involve pathological retinal neovascularization, blood–retinal barrier (BRB) and retinal thinning, and the degeneration of neurons, driven by multiple interrelated factors, including glucotoxicity, OS, inflammation, and hypoxia-driven growth factor secretion (87). LBP may simultaneously target these three key pathological processes, thereby exerting therapeutic effects of DR.

The vascular endothelial growth factor (VEGF) is a key inducer in stimulating the abnormal growth of new vessels in DR, while the pigment epithelium-derived factor (PEDF), as a protective factor, can inhibit angiogenesis (88). Yao et al. reported that compared with the DR group, LBP could increase the expression of PEDF by 1.5–1.8 times, reduce the VEGF content by 50–70%, and restore the balance between the two factors, thereby alleviating DM-stimulated pathological neovascularization (89). Furthermore, Liu et al. found that LBP could regulate the expression of miR-15a-5p and its downstream acid sphingomyelinase (ASM), VEGFA, vascular endothelial growth factor receptor 2 (VEGFR2), angiogenin 1 (ANG1), and ANG2, suppressing high glucose-induced RF/6A cell (monkey choroid-retinal endothelial cell line) proliferation and angiogenesis, ultimately delaying DR (90).

The BRB consists of the outer and inner BRB. The outer BRB is formed by retinal pigment epithelial (RPE) cells and their junctional complexes, while the inner BRB includes retinal vascular endothelial cells with tight junctions, supported by pericytes and Müller cells, collectively maintaining retinal microcirculation homeostasis (91). LBP was demonstrated to protect RPE cells against hyperglycemia- and H_2_O_2_-induced apoptosis through its antioxidant effect and to downregulate matrix metalloproteinase 2 (MMP2) derived from RPE cells, thereby jointly contributing to the outer BRB stabilization (92). Furthermore, LBP can also protect inner BRB by modulating the RhoA/RhoA-associated protein kinase 1 (RhoA/ROCK1) signaling pathway. In a DR rat model, LBP reversed the ROCK1 activation state of the endothelial cells, inhibited the activities of ROCK1 and phosphorylated myosin light chain (p-MLC), and upregulated the expression of p-Occludin, leading to a 30% reduction in Evans Blue (EB) infiltration volume, an indicator of BRB integrity and permeability, thus effectively alleviating retinal damage (93).

**Figure 6:**
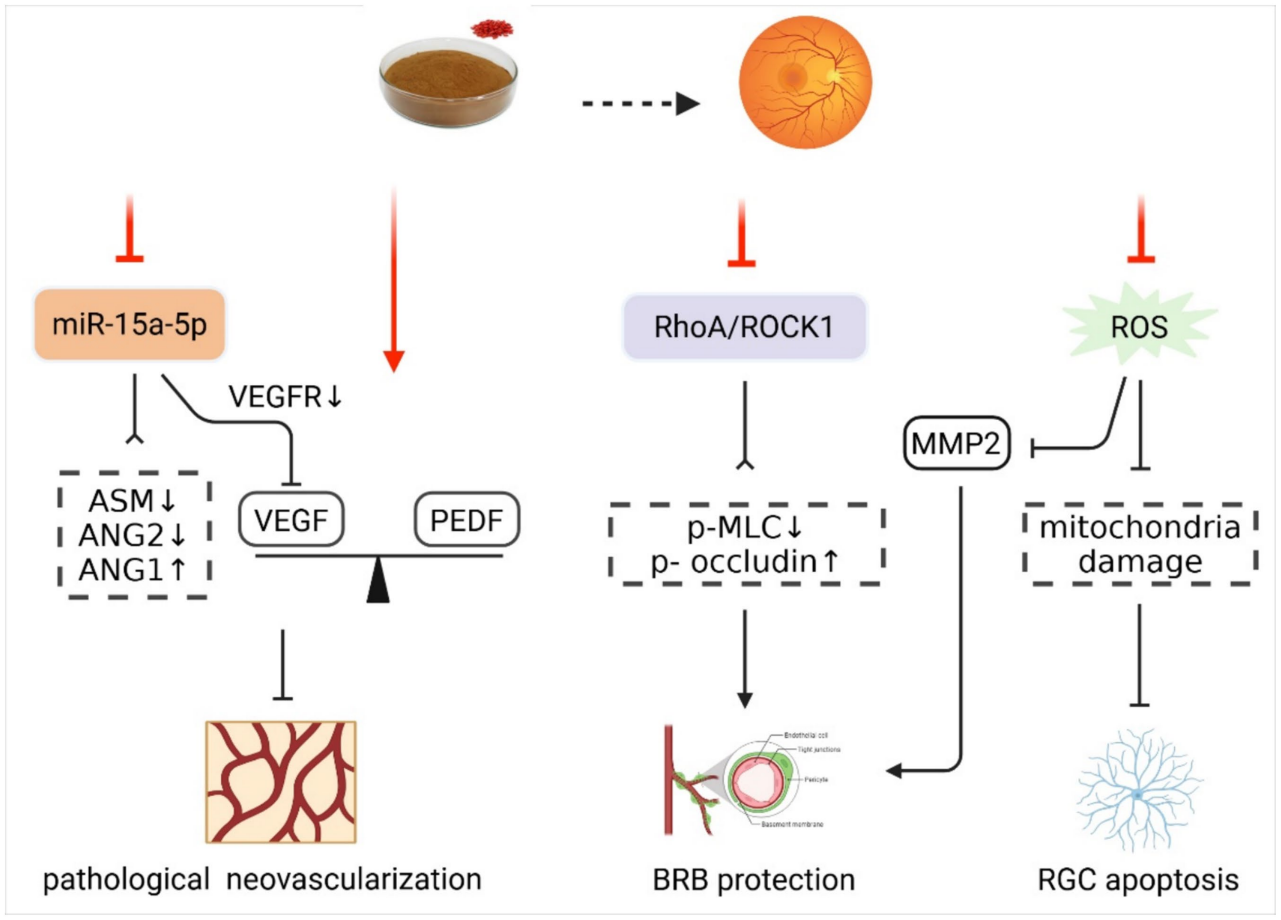
Effect and mechanism of LBP in treating DR. ANG1, angiogenin 1; ANG2, angiogenin 2; ASM, acid sphingomyelinase; BRB, blood-retinal barrier; MMP2, matrix metalloproteinase 2; PEDF, pigment epithelium-derived factor; p-MLC, phosphorylated myosin light chain; p-occludin, phosphorylated occluding; RGC, retinal ganglion cell; RhoA/ROCK1, RhoA/RhoA-associated protein kinase 1; ROS, reactive oxygen species; VEGF, vascular endothelial growth factor; VEGFR, vascular endothelial growth factor receptor. Created with BioRender.com.

Emerging evidence indicates that retinal neurodegeneration could be detected at the early stages of DR, even before significant microvessel and BRB alterations (94). Moreover, oxidative damage-induced apoptosis of retinal ganglion cell (RGC) mitochondria may contribute to the onset of DR (95). Notably, LBP has been shown to mitigate the pathological changes of mitochondria, protect RCG morphology and ultrastructure, inhibit neuronal apoptosis, and prevent the transition from nerve to vascular injury via its antioxidant properties, suggesting a potential therapeutic role in early-stage DR intervention (96) (Figure 6). In conclusion, LBP is not only a potent antioxidant but also a promising anti-VEGF agent and BRB protector, demonstrating substantial potential for the prevention and treatment of DR. These multifaceted protective effects warrant further clinical validation and mechanism exploration.

### 3.3 LBP and diabetic peripheral neuropathy

Diabetic peripheral neuropathy (DPN) is a form of hyperglycemia-induced peripheral nerve damage and dysfunction characterized by numbness, pain, swelling, and loss of sensation in the distal limb; > 50% of people with DM will develop into DPN (97). Notably, DPN severely impacts diabetic patients’ quality of life; it causes anxiety and depression, sleep disorders, and abnormal gait, and elevates the risk of diabetic wounds (98). LBP may alleviate DPN by improving OS, inhibiting endoplasmic reticulum stress (ERS) and apoptosis, promoting autophagy, and protecting myelin sheaths and Schwann cells. These mechanisms contribute to the amelioration of symptoms such as mechanical allodynia and thermal hyperalgesia, as well as improvements in motor and sensory nerve conduction velocities and increased amplitude of sensory nerve action potential (99–102).

The pathological basis of diabetic complications, including DPN, often involves oxidative damage from chronic hyperglycemia (103). In this context, Wu et al. demonstrated that LBP could exert a similar antioxidant effect as *α*-lipoic acid to regulate nerval GSH-Px and MDA levels, thereby attenuating nerve injury (99). Other than that, glucotoxicity-activated ERS can mediate neuronal apoptosis and nerve tissue damage, thereby promoting DPN progression (104). LBP has been proven to inhibit the protein kinase R-like endoplasmic reticulum kinase-CCAAT/enhancer-binding protein homologous protein (PERK-CHOP) pathway, the core regulatory mechanism of ERS, and then suppress the ERS-induced neuronal apoptosis, thus protecting neural function (100).

Autophagy, a cellular self-protection mechanism that removes damaged organelles and proteins via digestion, is crucial in maintaining cellular homeostasis and is regulated by the mammalian target of the rapamycin/p70 ribosomal protein S6 kinase (mTOR/p70S6K) signaling pathway (105). LBP could suppress the excessive activation of mTOR/p70S6K pathway to restore normal autophagy, upregulating myelin-related proteins such as myelin protein zero (P0) and myelin basic protein (MBP), and rescuing sciatic nerves’ myelin and axonal injury in DM rats (101).

**Figure 7:**
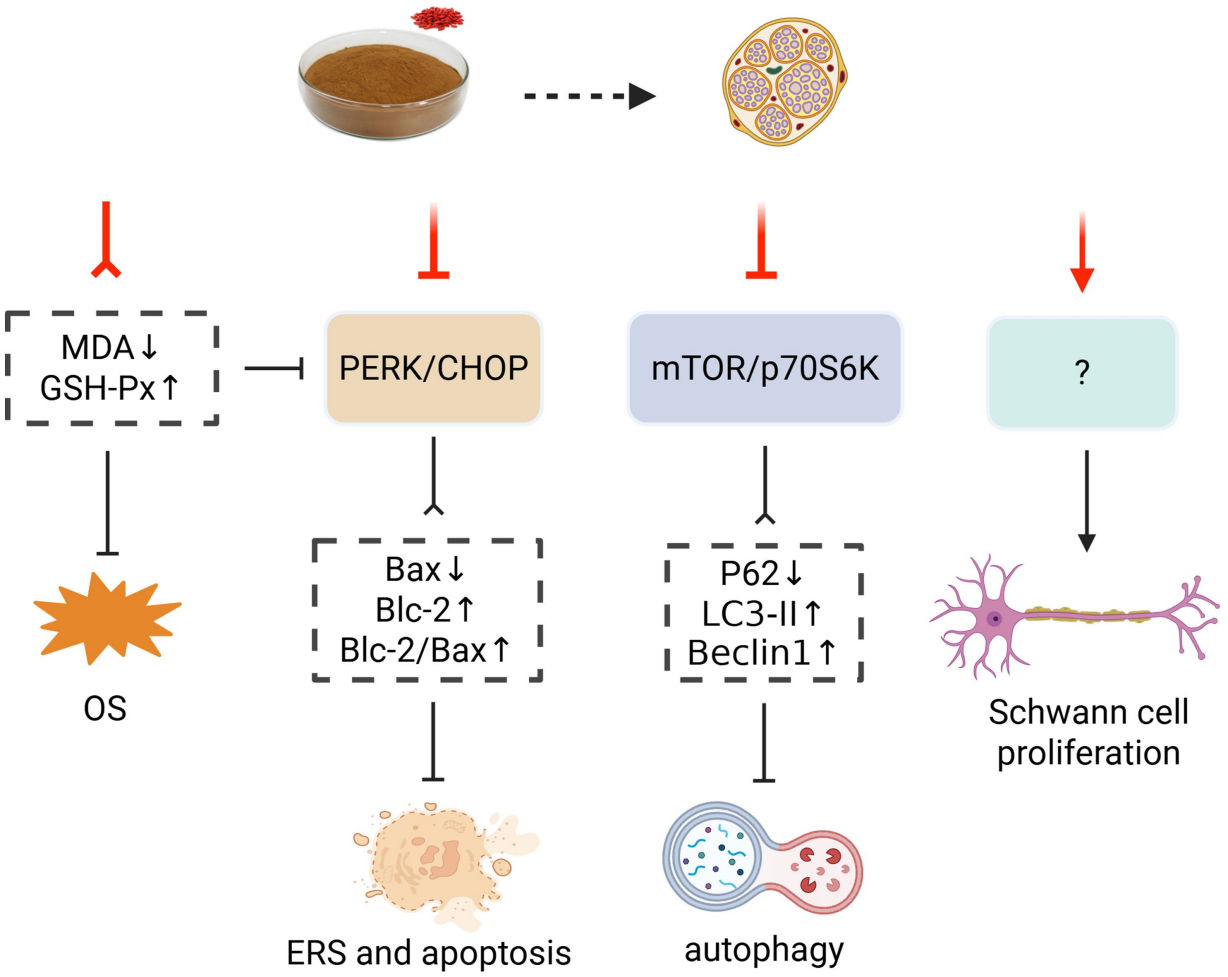
Effect and mechanism of LBP in treating DPN. Bax, Bcl-2-associated X protein; Bcl-2, B-cell lymphoma-2; ERS, endoplasmic reticulum stress; GSH-Px, glutathione peroxidase; LC3-II, light Chain 3 Protein II; MDA, malondialdehyde; mTOR/P70S6k, mammalian target of rapamycin/p70 ribosomal protein S6 kinase; OS, oxidative stress; P62, sequestosome 1; PERK/CHOP, protein kinase R-like endoplasmic reticulum kinase-CCAAT/enhancer-binding protein homologous protein. Created with BioRender.com.

Furthermore, Schwann cells—a type of glial cell, are crucial in maintaining neuronal structure and function, as well as in stimulating neuronal and myelin regeneration (106). Notably, Liu et al. reported that LBP dramatically elevated the viability of RSC96 cells (rat immortalized Schwann cell line) following high glucose incubation, although the potential mechanisms and biological effects require further exploration (102). This multi-mechanistic effect of LBP in improving DPN (Figure 7) warrants further validation in human studies, as well as subsequent development and integration into DM management strategies.

### 3.4 LBP and diabetic macroangiopathy

Diabetic macrovascular illnesses, including coronary artery disease (CAD), stroke, and peripheral artery disease (PAD), have been established to confer high cardiovascular death and disability risks in diabetic patients (107). The pathological basis of diabetic microangiopathy is traditionally an atherosclerosis (AS)-induced ischemic change (108). According to research, changes in the structure and function of large arteries and atherosclerotic plaque formation originate from hyperglycotoxicity-induced vascular endothelial cell (VEC) dysfunction (109). LBP was demonstrated to improve endothelium-dependent vasodilatation (EDV) in diabetic rats, with calpain-1 downregulation being a potential mechanism that may enhance endogenous nitric oxide synthase (eNOS) activity and increase nitric oxide (NO) content (110). Abnormal vascular smooth muscle cell (VSMC) proliferation and migration are the core steps in the progression to advanced vascular damage and AS (109). Fan et al. found that medium and high LBP concentrations inhibited the proliferation of high glucose-incubated A7r5 cells (rat thoracic aortic smooth muscle cell line); however, the specific molecular biological mechanism remained unclear, necessitating additional research (111). Additionally, LBP could stimulate the p38 MAPK signaling pathway and counteract the inflammatory response (e.g., reducing IL-6 and ICAM-1 levels) and OS (e.g., enhancing SOD and GSH-Px activities), alleviating the DM-induced vascular lesions (112). Overall, LBP can exert an ameliorative effect on the early onset of diabetic macrovascular disease.

In addition to being an independent risk factor for ischemic stroke onset and progression, DM might also worsen clinical outcomes by aggravating cerebral ischemia/reperfusion (I/R)-induced brain injury (113, 114). Besides preventing stroke by avoiding early damage of large arteries, LBP can also improve hyperglycemia-exacerbated cerebral I/R injury, impacting the prognosis of DM + stroke patients. In a hyperglycemia + transient middle cerebral artery occlusion (tMCAO) model, LBP significantly reduced cerebral edema, infarct volume, and neurological deficits scores, a phenomenon attributable to the alleviation of cerebral vascular remodeling and impaired vasoreactivity and neuronal death inhibition (115). Further study revealed that LBP relieved cerebral vascular endothelial and astrocyte endfeet swelling, reduced basement membrane protein and C-IV degradation, improved neurovascular unit dissociation, and upregulated TJ proteins (e.g., ZO-1, occludin, and claudin-5) in the same animal model (114). These findings suggest that LBP could ameliorate blood–brain barrier (BBB) leakage and structural damage, making it a therapeutic candidate for hyperglycemic stroke patients. Notably, ROS accumulation resulting from mitochondrial dynamics disorder could aggravate cerebral infarction. In this regard, LBP pretreatment was shown to regulate Opa1 and Drp1 proteins, balance mitochondrial fission/fusion, and protect the mitochondrial membrane structure, thus mitigating hyperglycemia-exacerbated cerebral I/R (116). In conclusion, LBP exerts direct protective effects on the vasculature and therefore holds promise for the prevention and treatment of diabetic macroangiopathy across the entire disease course.

### 3.5 LBP and diabetic cardiomyopathy

Diabetic cardiomyopathy (DCM) is a DM-induced impairment of cardiac structure and function in the absence of other cardiac risk factors (117). Under glycolipid toxicity and IR conditions, multiple mechanisms such as OS and inflammatory responses could be activated, leading to cardiomyocyte apoptosis, pathological ventricular remodeling, and cardiac microvascular diseases, potentially evolving into diastolic and systolic dysfunction, and ultimately developing into diabetic cardiac failure or even death (118).

Myocardial hypertrophy is a major manifestation of DCM. Calpain-1, a calcium-dependent cysteine protease, was reported to be overexpressed in a DM model (119, 120). Calpain-1 activation promoted the NF-κB signaling pathway to induce “prohypertrophic” genes in cardiomyocytes (121). Elsewhere, Calpain-1 was uncovered to stimulate the production of ROS in cardiomyocyte mitochondria, upregulate the expression level of adhesion molecules, activate acute inflammation, and promote the formation of myocardial hypertrophy (122). The therapeutic effect of LBP on STZ-induced DCM rats was documented previously (120), in which LBP abolished the STZ-induced myocardial damage, improved left ventricular systolic pressure (LVSP) and left ventricular end-diastolic pressure (LVEDP), reduced the ratio of heart weight/body weight (HW/BW), and suppressed the expression level of serum atrial natriuretic peptide (ANP) and brain natriuretic peptide (BNP), demonstrating that LBP may be an attractive treatment for myocardial hypertrophy and protect ventricular function. In the subsequent mechanistic study, it was observed that LBP reduced cardiac superoxide anion production and modulated the balance between eNOS and inducible nitric oxide synthase (iNOS). It also downregulated IL-6, TNF-*α*, ICAM-1, vascular adhesion molecule-1 (VCAM-1), and Toll-like receptor 4 (TLR4) protein levels. Furthermore, treatment with LBP suppressed the expression level of calpain-1 and suppressed the NF-κB nuclear translocation in myocardium of DCM rats. Collectively, these findings confirmed that LBP could treat DCM by inhibiting inflammation and OS, likely through the suppression of Calpain-1 expression and NF-κB activity. Evidence from prior investigations shows that elevated trimethylamine-N-oxide (TMAO) levels correlate with increased risk of cardiovascular diseases. Disruptions in GM composition and impairment of intestinal mucosal barrier can result in elevated circulating TMAO levels, which may contribute to myocardial injury through direct or indirect mechanisms (123). LBP could restore HFD-induced glycolipid metabolism and cardiac insufficiency by regulating GM-microbiome metabolite-host interactions, repairing intestinal barrier, and reducing serum TMAO concentration (124). These results suggest that LBP could also be a valuable adjunct therapy in managing DM-associated myocardial lesions.

### 3.6 LBP and diabetic cognitive dysfunction

Recent years have seen an ever-increasing prevalence of diabetic cognitive dysfunction (DCD), including mild cognitive impairment (MCI) and dementia, owing to the prolonged survival duration of diabetes patients (125). Clinically, DCD manifests as acquired cognitive and behavioral deficits that impair patients’ self-management capacity and treatment adherence, further accelerating disease progression (126). Research has shown that LBP can improve cognitive function in models of DCD or DM combined with Alzheimer’s disease (AD) rats. Zhao and Ye et al. found that LBP reduced the escape latency and increased the platform crossing times, target quadrant residence time and swimming distance in the Morris water maze test, which enhanced the learning and memory ability of diabetic rats (127, 128).

The available clinical guidelines suggest that the pathogenesis of DCD involves several processes, including metabolic disorders, abnormal cerebral insulin signaling, cerebrovascular endothelial damage, neuroinflammation, and neurodegenerative changes (126). GSK-3β is a critical kinase that modulates tau phosphorylation and functions as a downstream target of the Insulin/PI3K/AKT signaling pathway. In a diabetic brain, hyperglycemia and central IR can synergize to disrupt the PI3K/AKT/GSK-3β pathway, thereby triggering excessive GSK-3β activation, leading to hyperphosphorylation of the tau protein. Abnormal phosphorylation of the tau protein can induce the disintegration of microtubule structures and neuronal degeneration, causing cognitive impairment (129). A study showed that LBP exerted neuroprotective effects in hyperglycemic environment by regulating the PI3K/AKT/GSK-3*β* pathway, inhibiting tau protein hyperphosphorylation and enhancing axonal transport of injured neurons (127, 128). Moreover, data indicate that a high-fat, high-sugar diet and hyperglycemia-induced alterations in the gut environment may lead to DM-associated dysbiosis of GM. This DM-specific GM imbalance can increase gut permeability and systemic inflammation, negatively affecting brain function via the gut-brain axis (130). LBP, as a prebiotic polysaccharide, may preferentially correct the dysbiosis patterns unique to DM, to indirectly treat DCD. Tian et al. found that LBP regulated GM composition, protected intestinal barrier integrity, improved SCFAs secretion and associated GPCRs (e.g., GPCR41, GPCR43, and GPCR109A) expression in mice subjected to a high-fat and high-fructose diet (HFFD), thereby downregulating the release of IL-6, TNF-α, and LPS into circulation to alleviate neuroinflammation (131). Additionally, research has reported that sustained hyperglycemia promotes the formation of AGEs, which bind to their receptors on microglia, promoting their persistent activation and production of pro-inflammatory cytokines (132). Moreover, they further confirmed that LBP reduced the average fluorescence intensity of ionized calcium-binding adapter molecule-1 (Iba-1) and glial fibrillary acidic protein (GFAP), downregulated TNF-α and NOD-like receptor thermal protein domain-associated protein 3 (NLRP3) levels, and upregulated the expression of brain-derived neurotrophic factor (BDNF) and postsynaptic density protein-95 (PSD-95) expression in the hippocampus (131). Altogether, these findings provide compelling evidence that LBP can alleviate the HFFD-induced cognitive impairment by suppressing microglia activation and neuroinflammation. To sum up, oral supplementation with LBP could be a viable therapeutic approach for the management and/or avoidance of DCD.

### 3.7 LBP and diabetic foot ulcer

Diabetic foot ulcer (DFU) is one of the most serious complications of DM, characterized by the destruction of skin tissue caused by a combination of neuropathy, ischemia, and infection (133). DFU is increasingly considered a global issue owing to the development of refractory wounds, high disability and mortality rates (134). Strong chemokine response contributes to an overactive inflammatory response that inhibits neovascularization, leading to chronic non-healing wounds (135, 136). Previous studies demonstrated that LBP and LBP liposome nanoparticles (LBP-LNP) inhibit chemokine (CXC motif) ligand 12/chemokine (CXC motif) receptor 4 (CXCL12/CXCR4) signal transduction, prevent inflammatory response, enhance cellular autophagy, thereby stimulating metabolism and enhancing refractory wound repair in the diabetic foot (137). Nephrin is classically known as a podocyte protein in the kidney glomerular filtration barrier. Interestingly, recent findings indicate nephrin can also be expressed in epidermal keratinocytes in human skin and primary human epidermal keratinocytes (PHEKs) (138). These results suggest that keratinocyte nephrin may contribute to cell–cell adhesion and modulate keratinocyte migration and proliferation during the normal wound-healing process, possibly by regulating the nuclear translocation of yes-associated protein (YAP) and the organization of the actin cytoskeleton. Furthermore, hyperglycemia has been recognized to downregulate the expression of keratinocyte nephrin and impair keratinocyte migration, causing delayed wound-healing. Studies have shown that LBP and LBP-LNP can protect the structural integrity of foot epithelial cells and directly upregulate nephrin concentration in diabetic rat skin to alleviate delayed wound-healing under high glucose conditions (137). Collectively, these findings underscore the therapeutic potential of LBP in promoting diabetic wound healing.

## 4 Discussion and prospects

DM continues to be a major global public health issue with limited effective prevention and treatments. *In vitro* experiments and animal models showed that the plant-derived compound LBP could treat DM and its associated important chronic-complications, including DN, DR, DPN, diabetic macroangiopathy, DCM, DCD, and DFU. Although several plant polysaccharides have shown potential in combating DM, LBP is particularly recognized for its extensive effects and unique properties. For instance, compared with the common *Astragalus* polysaccharide (APS) and *Ganoderma lucidum* polysaccharides (GLP), LBP possesses a more potent antioxidant and anti-inflammatory effect, provides broader protection on pancreatic β-cells, and shows a stronger capacity to regulate the glycolipid metabolism disorder and gut-metabolism axis (132, 139, 140). Furthermore, LBP offers more extensive multi-organ protection and greater benefits in controlling complications. Given its strong benefits, easy availability, affordability, and fewer adverse effects, LBP can be used to develop alternative anti-diabetic therapies.

Although abundant and favorable preclinical evidence supports the use of LBP in the treatment of DM and its complications, the associated signaling networks warrant further investigation, particularly regarding the molecular mechanisms underlying its therapeutic effects, which remain to be fully elucidated. For instance, while the anti-renal fibrotic effects of LBP, as well as its protective effects on VSMCs and Schwann cells, have been confirmed, the signaling pathways mediating these effects require further clarification. Furthermore, as the global aging population continues to grow, more than 30% of DM patients are currently aged 60 years or older (141). Given the significant anti-aging properties of LBP, investigating its anti-diabetic mechanisms through the lens of cellular senescence, such as inhibition of macrophage senescence, represents a promising and scientifically compelling research direction (142). Moreover, emerging technologies and methods such as gene chips, single-cell sequencing, spatial transcriptomics, and organoid models should be utilized to comprehensively elucidate the multidimensional pharmacological mechanisms of LBP and reveal its potential for treating DM.

Furthermore, notwithstanding the growing body of preclinical evidence supporting the antidiabetic efficacy of LBP, substantial translational gaps remain that hinder its progression into clinical application. A key issue identified across the reviewed studies is the variability in the composition and lack of standardization of LBP extracts used. These investigations employed diverse extraction methods and purification techniques, resulting in heterogeneous LBP profiles, which complicate cross-study comparisons and limit translational relevance. It is therefore essential to elucidate the structure-activity relationship of LBP in the management of DM and to establish standardized protocols for polysaccharide production processes and quality control analyses. Future research should prioritize the development of eutherapeutic, well-characterized, and standardized preparations to ensure reproducibility and compliance with regulatory requirements in clinical settings.

LBP demonstrates multi-level and multi-target therapeutic actions against DM and its associated complications, with both overlapping and unique pathways across various disease states. Despite providing useful mechanistic insights and initial translational support through standardized animal models, existing preclinical studies face limitations in direct human applicability. This is primarily due to significant heterogeneity in outcomes, which can be traced to individual and species-specific differences, and a lack of methodological standardization in areas like dosing regimen and intervention duration. Given these challenges, we assert that a more rigorous and standardized approach to preclinical inquiry is paramount. Key priorities should include developing clinically representative animal models, incorporating interspecies pharmacokinetic considerations, and establishing consensus-driven methodological guidelines. These steps are vital for enhancing the translational value and reliability of DM research on LBP.

Given LBP’s hydrophilic nature, patient-friendly oral formulations such as capsules, granules, or liquid suspensions are viable options. However, due to its high degree of polymerization, large molecular weight, high viscosity, and poor solubility, LBP faces challenges of low oral bioavailability (143). To enhance its stability in the gastrointestinal tract and improve its oral bioavailability, advanced delivery systems should be explored. For example, encapsulation of LBP nanoparticles or liposomes can protect them from degradation and facilitate enhanced absorption, which is a critical prerequisite for efficacy (144). Furthermore, comprehensive pharmacokinetic analyses should be conducted in conjunction with pharmacodynamic data to establish a clear dose-response relationship. With regard to toxicological profiles, extensive studies affirm LBP’s safety. Acute and sub-chronic toxicity evaluations in both animal models and human trials show no significant adverse effects at therapeutic doses (145, 146). Nevertheless, more systematic and comprehensive toxicological evidence is required to define the specific toxicity and the safe dosage range clearly.

The currently available research highlights the paucity of clinical trial data regarding LBP’s antidiabetic properties, which presents an obstruction to its continued use. To date, only one clinical study has demonstrated the ability of LBP to improve clinical outcomes in DM patients. No clinical trials have yet been conducted to evaluate the pharmacological effects and safety profile of LBP in various complications of DM. Furthermore, there is a lack of systematic evaluation of the synergistic effects of LBP when used concomitantly with hypoglycaemic agents or other medications for diabetic complications. Therefore, additional well-designed, large-scale, and long-duration clinical studies are required to determine the short-term efficacy and long-term prognosis of LBP when used as monotherapy or in combination therapy for the management of DM and complications, thereby guiding clinical practice.

## 5 Conclusion

Overall, given the therapeutic potential and molecular mechanisms demonstrated in preclinical studies, it is worthwhile for researchers and product developers to consider LBP supplementation as a complementary strategy for the management of DM and its seven diabetic complications: DN, DR, DPN, diabetic macroangiopathy, DCM, DCD, and DFU. In light of current limitations in clinical translation, future research needs to focus on the key aspects, including LBP’s bioavailability, innovative dosage forms, safety and toxicology assessment, structure-activity and dose-effect relationship. Ultimately, standardized, multi-center, randomized, double-blind, placebo-controlled, or positive drug-controlled clinical trials should be conducted to rigorously assess and validate the clinical efficacy and safety of LBP on DM and its major complications, facilitating the translation of research findings into clinical practice.
